# Differentiation of site-specific symmetry breaking orders in Y_1−*x*_Pr_*x*_Ba_2_Cu_3_O_6+*y*_

**DOI:** 10.1038/s41467-026-72446-0

**Published:** 2026-04-29

**Authors:** Leonardo Martinelli, Sophie Rüdiger, Izabela Biało, Jens Oppliger, Fernando Igoa Saldaña, Martin von Zimmermann, Eugen Weschke, Riccardo Arpaia, Johan Chang

**Affiliations:** 1 Physik-Institut, Universität Zürich, Zürich, Switzerland; 2 Deutsches Elektronen-Synchrotron DESY, Hamburg, Germany; 3 Helmholtz-Zentrum Berlin für Materialien und Energie, Berlin, Germany; 4 Department of Molecular Sciences and Nanosystems, Ca’ Foscari University of Venice, Venice, Italy; 5 Quantum Device Physics Laboratory, Department of Microtechnology and Nanoscience, Chalmers University of Technology, Göteborg, Sweden

**Keywords:** Superconducting properties and materials, Electronic properties and materials

## Abstract

Solid matter is classified through symmetry of ordering phenomena. Experimentally, this approach is straightforward, except when distinct orderings occur with identical or almost identical symmetry breaking. Here we show that the cuprate system Y_1−*x*_Pr_*x*_Ba_2_Cu_3_O_6+*y*_ hosts two distinct orderings with almost identical translational symmetry breaking. Only when applying site-sensitive resonant elastic x-ray scattering, charge ordering can be conclusively differentiated from a super-lattice structure. These two orderings manifest at different atomic sites and display different temperature dependence. Differentiating these orders provides an important clue to the anomalous behavior of PrBa_2_Cu_3_O_7_ within the 123-series of high-temperature superconductors. The superstructure symmetry breaking at the Pr-site should be included into future models of the unusual insulating ground state of PrBa_2_Cu_3_O_7_.

## Abstract (web summary)

Classification of ordering phenomena can be challenging when distinct orderings occur with identical or almost identical symmetry breaking. Here, the authors identify two distinct orderings with almost identical translational symmetry breaking in Y_1−*x*_Pr_*x*_Ba_2_Cu_3_O_6+*y*_.

## Introduction

The high-temperature superconducting compound family REBa_2_Cu_3_O_6+*y*_ (REBCO) is used to enhance the performance of critical technologies such as windmill rotors^1^, fusion tokamaks^2^, and magnets^3^, including MRI^4^ devices. Generally, these compounds display similar superconducting onset temperatures and magnetic ordering at very low temperatures—irrespective of the rare-earth (RE) element. However, PrBa_2_Cu_3_O_6+*y*_ (PBCO) represents a puzzling anomaly. Although maintaining the same average crystal structure, PBCO is not superconducting or even metallic. Moreover, the Pr ions order antiferromagnetically at temperatures one order of magnitude larger (~12–17 K) than other RE ions^5^. Therefore, PBCO has been the subject of theoretical scrutiny. The most accepted theoretical model that explains the suppression of superconductivity was developed by Fehrenbacher and Rice (FR)^6^. It revolves around the hybridization of the Pr-4*f* and O-2*p* orbitals, which—in turn—impedes efficient doping of the CuO_2_ planes. The FR model considers a local hole state formed by the coordination of O-2*p*_*π*_ orbitals oriented towards the Pr site with $4{f}_{z({x}^{2}-{y}^{2})}$ symmetry. The result is a $4{f}^{1}+4{f}^{2}\underline{L}$ Pr configuration ($\underline{L}$ stands for a hole in the oxygen ligand bands), so that Pr has a mixed 3^+^/4^+^ valence. This model was able to explain some of the experimental results, including inelastic neutron scattering, X-ray absorption, and magnetic susceptibility^7,8^.

An important question relates to electronic ordering in PBCO. Charge ordering^9–25^ and associated electronic reconstruction^26–29^ have been studied in great detail in YBa_2_Cu_3_O_6+*y*_ (YBCO). Generally, a two-dimensional, bi-directional charge-density-wave (CDW) order is found in a doping range centered around the so-called 1/8-anomaly^30–32^. Upon application of magnetic field^33,34^ or uniaxial pressure^35^, a three-dimensional unidirectional charge order was reported. The two types of CDW structures are linked as they occur with exactly the same in-plane ordering vector. Furthermore, both orders seem to compete with superconductivity. More recently, evidence of unexpected orders has been given in compounds with partial Y→Pr substitution using resonant soft X-ray scattering^36,37^. The additional orders have been interpreted as a modulation similar to CDW related to Pr electrons, although only mildly changing temperature. Based on its wavevector, it has been interpreted as an electronic charge density wave. The picture is, at present, however, largely incomplete and partially contradictory. The order has been reported in compounds with different Pr substitution (0.3 in ref. ^36^ and ≥0.8 in ref. ^37^), at different out-of-plane wavevectors (*L* = 1^36^ and *L* = 1.5^37^), and with different temperature dependences.

Here, we present a comprehensive study of PrBa_2_Cu_3_O_7_ and Y_1−*x*_Pr_*x*_Ba_2_Cu_3_O_6+*y*_ (*x* = 0.3, *y* = 0.67, and 1) relaxed films. Lattice and charge modulations are investigated by a combination of high-energy grazing incidence X-ray diffraction (GI-XRD) and resonant elastic X-ray scattering (REXS) (more precisely referred to as energy-integrated resonant inelastic X-ray scattering). Across the (*x*, *y*) phase diagram, we identify two ordering vectors: *Q*_1_ = (*δ*_1_, 0, 0), only present with Pr doping and absent in stoichiometric YBCO, and *Q*_2_ = (*δ*_2_, 0, 1/2) with *δ*_1_ ≈ *δ*_2_ ≈ 1/3. Although these two orders display similar in-plane modulation wavevectors, we demonstrate that they originate from different mechanisms. (1) They resonate at different atomic sites. *Q*_1_ resonates at the Pr and out-of-plane copper sites, whereas *Q*_2_ resonates at the in-plane copper site. (2) They display different temperature dependences, as the *Q*_2_-order disappears above 250 K, whereas the *Q*_1_ one is essentially temperature independent. (3) The two orders display different correlation lengths: *Q*_1_ is a long-range order with >100 Å, whereas *Q*_2_ has a much shorter correlation length. (4) The in-plane periodicity shows a minute discrepancy, in that we find *δ*_2_ < 1/3 < *δ*_1_. We interpret the *Q*_2_ order in terms of a charge-density-wave, identical to what has been reported in YBa_2_Cu_3_O_6+*y*_. Instead, based on the intensity of the peak over multiple zones, we provide strong evidence that the *Q*_1_ order is, in contrast to refs. ^36,37^, a Pr-related super-lattice structure. We demonstrate that the low intensity of the peak in the first Brillouin zone, which in refs. ^36,37^ is comparable to that of standard CDW, which is a structure factor effect. We argue that this additional superstructure must be incorporated to understand the unusual electronic properties of PBCO.

## Results

### Non-resonant X-ray scattering

**Fig. 1:**
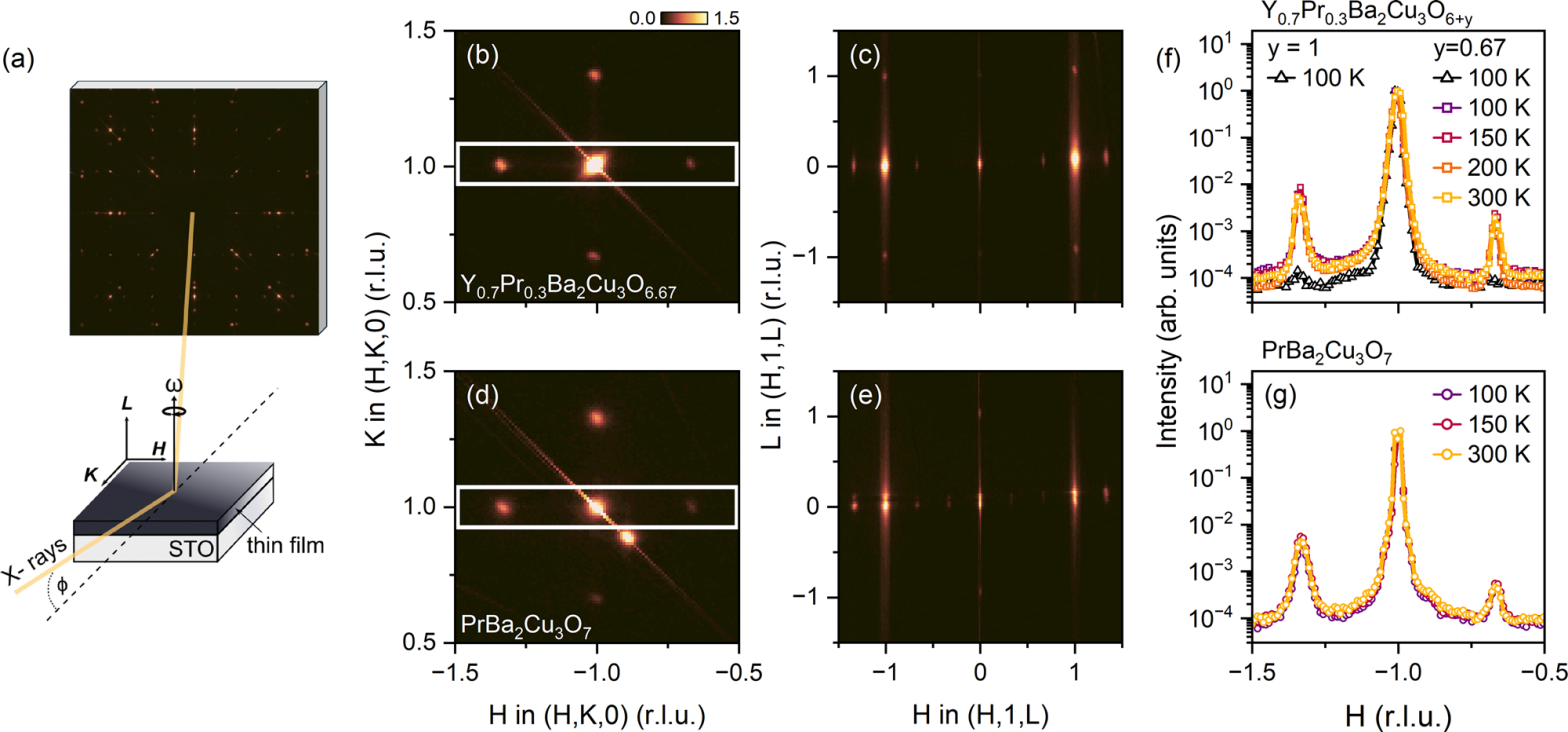
Grazing-incidence X-ray diffraction on PrBa_2_Cu_3_O_7_ and Y_0.7_Pr_0.3_Ba_2_Cu_3_O_6.67_ thin films. **a** Schematic illustration of the scattering technique. The X-ray incidence angle *ϕ* has been kept fixed. The films are rotated continuously by the angle *ω* around the axis normal to the film surface, while detector images are recorded at a fixed framerate. The actual value of *ω* is measured for each frame. **b**–**d** Scattering volumes—represented by the (*H*, *K*, 0) and (*H*, 1, *L*) scattering planes—recorded on Y_0.7_Pr_0.3_Ba_2_Cu_3_O_6.67_ (**b**, **c**) and PrBa_2_Cu_3_O_7_ (**d**, **e**) thin films. The scattering intensity is displayed with a logarithmic false color scale. **f**, **g** One-dimensional intensity scans along (*H*, 1, 0) derived from the data shown in (**b**, **d**) and for temperatures as indicated, normalized to the intensity of the (−1, 1, 0) Bragg reflection. In (**c**), a scan recorded on Y_0.7_Pr_0.3_Ba_2_Cu_3_O_7_ is included (black triangles).

The combination of the high-energy X-ray diffraction with grazing-incidence geometry provides a reciprocal space overview with high sensitivity to the sample surface layers^38^. In this work, we take a further step and combine high-energy grazing-incidence diffraction with low temperatures to investigate structural orderings in relaxed films of PrBa_2_Cu_3_O_7_, Y_0.7_Pr_0.3_Ba_2_Cu_3_O_7_, and Y_0.7_Pr_0.3_Ba_2_Cu_3_O_6.67_. In Fig. 1, (*H*, *K*, 0) and (*H*, 1, *L*) scattering planes are obtained by slicing the three-dimensional reciprocal scattering volume. The most intense reflections are associated with fundamental Bragg peaks at *Q*_*B*_ = (*H*, *K*, *L*) with integer Miller indices. Furthermore, quasi-commensurate weaker reflections are found at *Q*_1_ = *Q*_*B*_ + *τ*_1_ with *τ*_1_ ≈ (±*δ*_1_, 0, 0) and (0, ± *δ*_1_, 0) where *δ*_1_ ≿ 1/3. These quasi-commensurate reflections are two to four orders of magnitude weaker than the fundamental Bragg reflections in PrBa_2_Cu_3_O_7_ and Y_0.7_Pr_0.3_Ba_2_Cu_3_O_6.67_—see Fig. 1c, e. In Y_0.7_Pr_0.3_Ba_2_Cu_3_O_7_, these reflections are yet another one to two orders weaker and are only observed in a few Brillouin zones. In all cases, the *Q*_*B*_ and *Q*_1_ reflections are essentially temperature independent in the explored temperature range from 100 to 300 K (see Fig. 1c). The correlation length of the *Q*_1_ reflection also appears to be temperature independent. After estimation of the experimental resolutions of the GI-XRD and REXS (see Supplementary Material), we extract a momentum full-width-at-half-maximum (FWHM) of ~0.015 (0.01) r.l.u. for GI-XRD (REXS) for Y_0.7_Pr_0.3_Ba_2_Cu_3_O_6.67_, and of 0.022 (0.014) r.l.u. for PrBa_2_Cu_3_O_7_. The discrepancy between the two sets of measurements can be explained by the fact that GI-XRD probes the whole volume of the films, while REXS is limited to a spot size of 100 × 50 μm. This corresponds to a spatial correlation length $\xi \simeq \frac{2}{\,{{\rm{FWHM}}}}$ Å ~ 170 Å using the estimate from REXS.

### Site-sensitive measurements

**Fig. 2:**
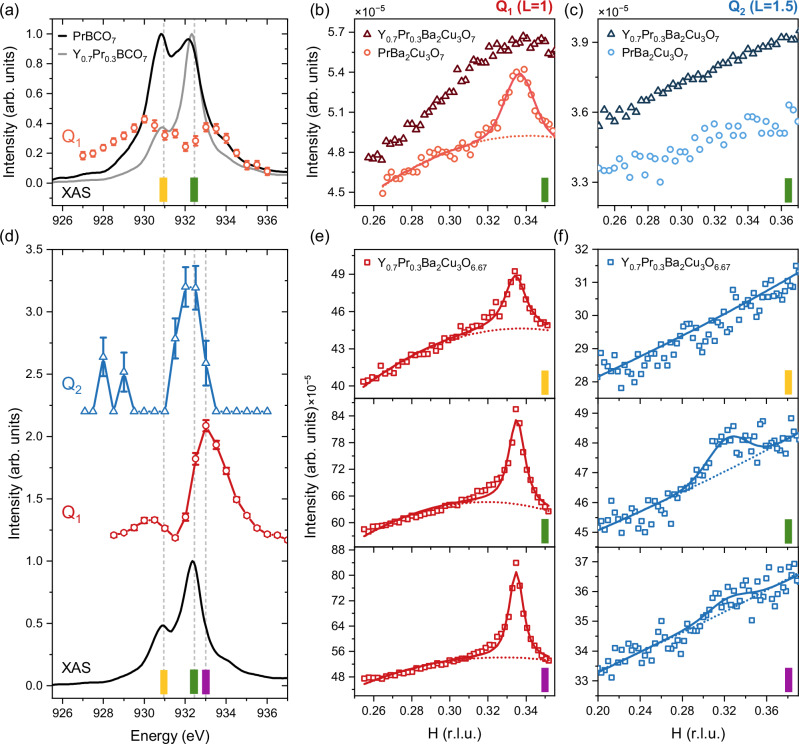
X-ray absorption spectroscopy (XAS) and resonant elastic X-ray scattering (REXS). **a**–**c** XAS and REXS on PrBa_2_Cu_3_O_7_ and Y_0.7_Pr_0.3_Ba_2_Cu_3_O_7_. **a** XAS for PrBa_2_Cu_3_O_7_ (black) and Y_0.7_Pr_0.3_Ba_2_Cu_3_O_7_ (gray), the Pr-*M* and Cu-*L* resonances (indicated by vertical dashed lines). The REXS signal at (1/3, 0, 1) and (1/3, 0, 1.5) is shown with red and blue points, respectively. Scans on PrBa_2_Cu_3_O_7_ are indicated with circles, on Y_0.7_Pr_0.3_Ba_2_Cu_3_O_7_ with triangles. **d**–**f** XAS and REXS on Y_0.7_Pr_0.3_Ba_2_Cu_3_O_6.67_. **d** XAS and energy dependence of *Q*_1_ and *Q*_2_ reflections (the amplitudes of *Q*_1_ and *Q*_2_ are not in scale). **e**, **f** Momentum dependence of the REXS signal through (1/3, 0, 1) and (1/3, 0, 1.5) at, respectively, the Pr resonance (930.95 eV, yellow mark), in-plane Cu-*L* resonance (932.45 eV, green mark), and out-of-plane Cu-*L* resonance (933 eV, purple mark). In the REXS momentum scans, solid lines represent Gaussian fits to the data, and dashed lines indicate linear or quadratic background. The fit in (**f**) of the out-of-plane peak indicate weak peak, which, however, is not statistically significant. All curves were measured at *T* = 10 K. Error bars in (**a**, **d**) are estimated as 95% confidence intervals on fitting parameters.

Resonant scattering experiments were carried out on both the Cu-*L* (932.1 eV) and Pr-*M* resonances (930.9 eV), as shown in Fig. 2a. The labeling of these two closely lying resonances is reached by varying the Pr concentration. The first peak in the XAS resonance clearly scales with Pr content—see Fig. 2a, d—while the second feature at 930.95 eV shows less dependence on the Pr content. The Pr resonance is likely complex and composed of multiple peaks. Previous XANES experiments^7^ suggested a Pr valence of ~3.2, so that a 20% contribution of Pr^4+^ states is expected. The Pr^4+^ contribution peaks around 1 eV higher than Pr^3+^ and has a broad lineshape (see ref. ^39^), so that it partially overlaps with the Cu white line. As revealed by the non-resonant grazing incident diffraction, the crystal structure includes a *τ*_1_ modulation. With resonant scattering, the quasi-commensurate reflection is probed through *Q*_1_ = (*δ*_1_, 0, 1). In this zone, the reflection is too weak to be observed in Y_0.7_Pr_0.3_Ba_2_Cu_3_O_7_, but intense reflections are found in PrBa_2_Cu_3_O_7_ and Y_0.7_Pr_0.3_Ba_2_Cu_3_O_6.67_. The photon-energy dependence of this commensurate peak displays maxima at the Pr^3+^ and out-of-plane Cu (and possibly Pr^4+^) resonances (see Fig. 2a, b). The peak positions—determined through Gaussian fits—are, respectively, *δ*_1_ = 0.3365(4) for Y_0.7_Pr_0.3_Ba_2_Cu_3_O_6.67_ and *δ*_1_ = 0.3360(5) for PrBa_2_Cu_3_O_7_. Thus, for both compounds *δ*_1_ > 1/3. Just as with non-resonant scattering, these reflections are essentially temperature independent.

### Charge density wave order

**Fig. 3:**
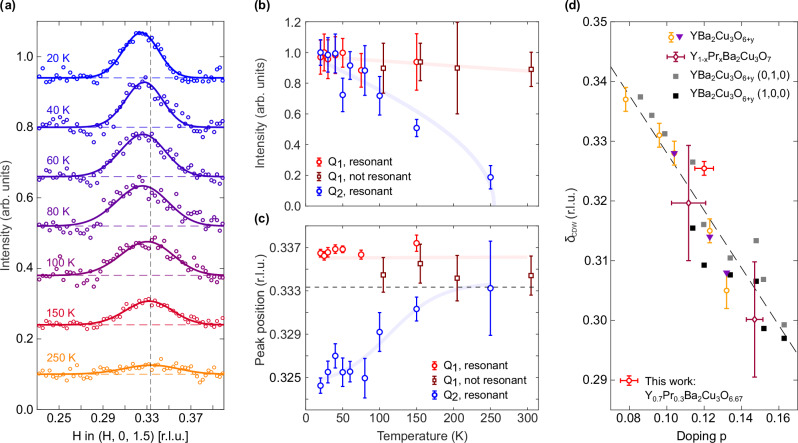
Temperature dependence of the charge order in Y_0.7_Pr_0.3_Ba_2_Cu_3_O_6.67_. **a** Background subtracted intensity versus momentum across the charge ordering vector in Y_0.7_Pr_0.3_Ba_2_Cu_3_O_6.67_, for temperatures as indicated. Solid and dashed lines are, respectively, Gaussian fits and the linear background. **b** Intensity (amplitude) of the charge-density-wave order reflection (blue) compared with the *L* = 0 modulation. **c** Incommensurabilities of the two modulations found in Y_0.7_Pr_0.3_Ba_2_Cu_3_O_6.67_ as a function of temperature. XRD results are an average over multiple zones as explained in Section S4 of the Supplementary Material. Error bars in (**b**, **c**) indicate standard deviations of the Gaussian fits. Shaded lines in (**b**, **c**) are guides to the eye. **d** Charge-density-wave incommensurability in YBa_2_Cu_3_O_6+*y*_ from refs. ^12^ (yellow dots),^13^ (purple dots),^17^ (black and gray squares), and Y_1−*x*_Pr_*x*_Ba_2_Cu_3_O_6+*y*_^37^ (dark red dots). Dashed lines are guides to the eye. Error bars for refs. ^12,13,17,37^ are taken from the corresponding works. Doping error bars for our data (red point) are estimated from the broadness of uperconducting transition (see Supplementary Material^53^).

Only in Y_0.7_Pr_0.3_Ba_2_Cu_3_O_6.67_, a much weaker reflection is found at *Q*_2_ = (*δ*_2_, 0, 1/2) with *δ*_2_ = 0.325(1) < 1/3. This reflection is most intense at the in-plane Cu-*L* resonance (Fig. 2b). Furthermore, it displays a significant temperature dependence, as shown in Fig. 3a, and essentially disappears at 250 K. The peak amplitude—quantified through Gaussian fits—decreases with increasing temperature (Fig. 3b). At high temperature (with weakened amplitude), the peak position slightly shifts to larger momentum (Fig. 3c)—as previously observed in La- and Bi-based cuprates^40,41^. Importantly, the *Q*_2_ reflection shows little dependence on the out-of-plane momentum *L* (Fig. S4 in the Supplementary Material), indicating a predominant two-dimensional character. These evidences suggested that the observed reflection is the usual charge order observed in underdoped, hole-doped cuprates. The FWHM shows a typical trend for charge order, decreasing with temperature and plateauing below the onset of superconductivity, as shown in Fig. S3 in the Supplementary Material. The maximum correlation length, calculated as $\frac{a}{\pi \cdot \,{{\rm{FWHM}}}}$, is around 30 Å. This value is approximately two times smaller than the correlation length in YBa_2_Cu_3_O_6.67_^17^. We also stress that, compared to YBCO_6.67_ (*p* = 0.12), the (static) charge density wave peak has a significantly higher onset temperature. Yet, the low-temperature commensuration is consistent with that of charge-density-wave order found in YBCO for the same oxygen concentration (Fig. 3d).

## Discussion

**Fig. 4:**
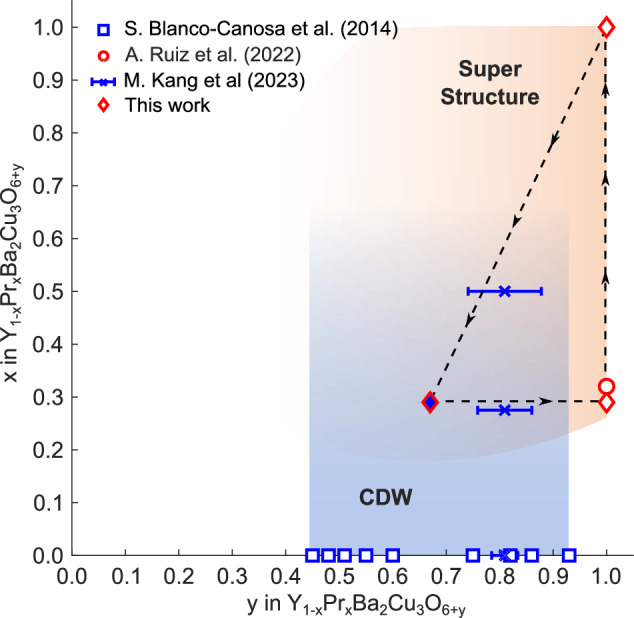
Structural phase diagram of Y_1−*x*_Pr_*x*_Ba_2_Cu_3_O_6+*y*_. Charge order (blue) and super-lattice structure (orange) depend on oxygen content and Pr substitution. Color-shaded areas schematically show the extent of the two phases. Red and blue symbols indicate, respectively, the observation of *Q*_1_ = (*δ*_1_, 0, 1)-order (herein interpreted as a super-structure) and *Q*_2_ = (*δ*_2_, 0, 1/2) charge density wave order. The figure includes previous studies (refs. ^36,37^), and Pr- and O- content are inferred from them.

In YBa_2_Cu_3_O_6+*y*_, charge orders with integer and half-integer out-of-plane modulation have been observed with exactly the same in-plane ordering vector^33,34,42^. A schematic phase diagram of the different peaks observed in this study and recent literature (refs. ^36,37^) as a function of oxygen content and Pr substitution is reported in Fig. 4. Due to similar in-plane periodicities, these orders have been interpreted in terms of different stacking patterns of the same in-plane charge-density wave order. The observation, in Y_1−*x*_Pr_*x*_Ba_2_Cu_3_O_6+*y*_, of integer and half-integer out-of-plane modulations with almost the same in-plane ordering vectors is reminiscent of what has been found in YBCO^36^. However, there are at least three important differences. (i) Although close, the in-plane ordering vectors (in Y_0.7_Pr_0.3_Ba_2_Cu_3_O_6.67_) are not identical. (ii) The two orders are resonating at different atomic sites. (iii) The integer out-of-plane ordering displays no temperature dependence. For these reasons, we will discuss the two orderings separately.

The chemical inclusion of partial Pr-substitution may influence the distribution of doped holes. In YBCO, an oxygen content of 6.67 corresponds to 0.12 holes per in-plane copper site^43,44^. Generally, we label *p*_ZR_ for the hole concentration within the CuO_2_ planes. By contrast, holes hybridizing between oxygen and Pr sites are labeled with *p*_FR_. In this fashion, we consider both Zhang-Rice and Fehrenbacher-Rice configurations. The sum *p*_ZR_ + *p*_FR_ is bounded by the total hole doping *p*—controlled by the oxygen content. The relatively low superconducting transition temperature (*T*_*c*_ = 30 K) in our Y_0.7_Pr_0.3_Ba_2_Cu_3_O_6.67_ thin film may indicate a reduced hole doping *p*_ZR_ within the CuO_2_ planes. The remaining holes *p*_FR_ would be residing within the Pr 4*f*-FR state. The charge-density-wave incommensurability suggests a hole doping of *p*_ZR_ ≈ 0.10 within the CuO_2_ planes. As such, our results suggest that only a small fraction of holes (*p*_FR_ = 0.02) goes into the Pr 4*f*-FR state, and that these holes do not participate in the charge order modulation.

The weak reflection occurring at *Q*_2_ = (*δ*_2_, 0, 1/2) in Y_0.7_Pr_0.3_Ba_2_Cu_3_O_6.67_is here interpreted as evidence of charge-density-wave order. Phenomenologically, this order resembles the two-dimensional charge-density-wave order observed in YBCO. It has the same ordering vector, resonates at the in-plane copper site, and shows a similar decrease with temperature. The larger onset temperature of charge-density-wave order combined with a lower superconducting transition temperature could be interpreted in terms of phase competition. Below the superconducting critical temperature, a saturation of the charge-density wave intensity is observed, similar to that in other cuprates^45^.

Next, we turn to the *Q*_1_-order manifested by quasi-commensurate peaks at *Q*_1_ = (*δ*_1_, 0, 0). For PrBa_2_Cu_3_O_7_, *δ*_1_ > 1/3, whereas charge-density-wave ordering in optimally doped YBCO has an incommensurability *δ*_2_ < 1/3^13,17^. The diffraction intensity of the *Q*_1_ peaks and the absence of temperature dependence, furthermore, are at odds with the expectations for an electronic order. In what follows, we discuss this ordering in terms of a super-lattice structure. Given that this ordering resonates at the out-of-plane Cu and Pr sites, this suggests that it is not linked to the in-plane Cu orbitals. We observe this super-lattice structure in both PrBa_2_Cu_3_O_7_, Y_0.7_Pr_0.3_Ba_2_Cu_3_O_7_, and Y_0.7_Pr_0.3_Ba_2_Cu_3_O_6.67_. In Y_0.7_Pr_0.3_Ba_2_Cu_3_O_7_ and Y_0.7_Pr_0.3_Ba_2_Cu_3_O_6.67_, the 1/3 partial substitution provides a direct motive for such a superstructure. A Pr-substitution every third unit cell would generate peaks at *Q*_1_. Given that the *Q*_1_ order manifests strongest in PrBa_2_Cu_3_O_7_ and weakest in Y_0.7_Pr_0.3_Ba_2_Cu_3_O_7_, this suggests that the inclusion of Pr in general generates a susceptibility for a 1/3 super-lattice structure.

Next, we discuss the phenomenological differences with refs. ^36,37^. In particular, ref. ^36^ reports a *Q*_1_ peak in a bulk crystal of Y_0.7_Pr_0.3_Ba_2_Cu_3_O_7_ with a much higher intensity than what we observe in our corresponding film. This discrepancy might be related to the reduced structural coherence of the Pr-induced modulation in thin films, where epitaxial strain, higher defect density, and finite thickness are known to weaken long-range ordering of subtle structural modulations (as observed, for instance, for the CuO-chain order in YBCO films). An additional contribution may arise from small deviations in the actual Pr content of thin films relative to the nominal composition. In pulsed laser deposition (PLD) growth, different sticking coefficients and the higher volatility of Pr compared to Y can lead to an effective substitution level on the Y site that slightly deviates from the target stoichiometry. ref. ^37^, on the other hand, reports the presence of two Pr- and Cu-resonating peaks at half-integer *L* = 1.5 in PrBa_2_Cu_3_O_7_, and a single weak CDW peak in Y_1−*x*_Pr_*x*_Ba_2_Cu_3_O_6+*y*_ for *x* ~ 0.3 (with *y* ~ 0.8 estimated by the authors). This last result is in agreement with our *Q*_2_ order. However, neither this work nor ref. ^36^ find the presence of any Pr-related or Cu-related orders in PrBa_2_Cu_3_O_7_at *L* = 1.5. We can speculate that the small oxygen deficiency *y* ~ 6.8 of the films used in the work may play a role, as well as the smaller thickness of the films employed (40 nm). It would also be of great interest to investigate whether the samples used in ref. ^37^ show the presence of any structure at integer *L*-positions. In general, the broad phenomenology is similar in the three studies. Therefore, we believe that, despite discrepancies which might be related to differences in sample type (thin films vs single crystals), the core phase diagram presented in Fig. 4 is generally valid for the Y_1−*x*_Pr_*x*_Ba_2_Cu_3_O_6+*y*_system.

As PrBa_2_Cu_3_O_7_ is a stoichiometric compound, structure refinement is, in principle, possible from the large reciprocal scattering volume collected. We leave such a detailed analysis for future communication. Instead, we bring forward symmetry arguments and the associated symmetry allowed distortion modes *u*_*i*_. Given that the ordering resonates at the Pr site, this atomic site is certainly involved in the super-lattice structure. The *Q* = (*δ*_1_, 0, 0) ordering implies an in-plane distortion *u* = (*u*_*x*_, 0, 0, ) of the Pr atoms. Given the average *Pmmm* crystal structure of PrBa_2_Cu_3_O_7_, the ordering vector allows four possible subspace groups—identified through the Isodistort software package^46^ (see Supplementary Table II). From our diffraction data, it is not possible to differentiate between bi-axial and twinned uniaxial orderings. Irrespectively of the order parameter symmetry, orthorhombic symmetry allowed space groups are *Pmmm* and *Pmm2*. For bi-axial symmetry, two additional monoclinic subspace groups are allowed (*P2/m* and *Pm*). In all cases, in-plane Pr distortion modes are present. The causal relation between Pr-site distortions and mixed Pr-valence from oxygen hybridization remains to be clarified. It is, however, clear that the observed *Q*_1_-order will influence the hybridization with in-plane oxygen orbitals. As such, the reported super-structure links directly to the anomalous electronic properties of PrBa_2_Cu_3_O_7_. We conclude that translational symmetry breaking at the Pr-site is detrimental to superconducting pairing. In perspective, although Scanning Tunneling Microscopy remains challenging for these samples (and in general for the REBCO family), progress in surface preparation and termination control could make real-space, site-sensitive measurements increasingly accessible, further clarifying the microscopic origin of the observed *Q*_1_ order.

## Methods

### Film growth

Fully oxygenated, insulating PrBa_2_Cu_3_O_7_ (PBCO) films, 90 nm thick, were deposited on 5 × 5 mm^2^ (001)-oriented SrTiO_3_ substrates by radio-frequency (rf) sputtering. The deposition was carried out at a power of 50 W, a total pressure of 0.1 mbar (with an Ar:O_2_ ratio of 4:1), and a substrate temperature of 800 °C. The Y_1−*x*_Pr_*x*_Ba_2_Cu_3_O_6+*y*_ (Pr-YBCO) films, with a thickness of 120 nm, were grown on similar 5 × 5 mm^2^ (001) SrTiO_3_ substrates using PLD at a heater temperature of 760 °C and an oxygen pressure of 0.6 mbar. After deposition, the films were slowly cooled to room temperature in an oxygen pressure of 700 mbar to ensure full oxygenation, resulting in Pr-YBCO with *y* = 1^47^. To obtain underdoped Pr-YBCO films with reduced chain oxygen content (*y* = 0.67), fully oxygenated samples were subjected to an ex situ annealing process^48^. This consisted of 9 h of treatment at 550 °C in a reduced oxygen atmosphere of 0.02 mbar. The superconducting critical temperatures, *T*_c_, were determined from resistance versus temperature measurements, *R*(*T*), performed using a PPMS DynaCool system (Quantum Design). The films exhibited *T*_c_ values of 53 K and 28 K for *y* = 1 and *y* = 0.67, respectively. Structural characterization was carried out using X-ray diffraction. The out-of-plane reflections were used to extract the *c*-axis lattice parameter, which, combined with *T*_c_, was used to estimate the oxygen content *y*^41,47^. In-plane diffraction measurements confirmed the presence of the twinned orthorhombic *Pmmm* crystal structure, consistent with the symmetry of the SrTiO_3_ substrates, and indicative of the absence of long-range chain order in the films. Since the substrate has a tetragonal structure, all the investigated films present an almost perfect twinning. The lattice constants and thicknesses of the investigated films are reported in Table 1.

**Table 1:** Thickness *d* and lattice parameters for the films, all grown on STO, studied in this work

Compound	*d* [nm]	*a* [Å]	*b* [Å]	*c* [Å]
PrBa_2_Cu_3_O_7_	90	3.895	3.895	11.82
Y_0.7_Pr_0.3_Ba_2_Cu_3_O_7_	60	3.858	3.907	11.65
Y_0.7_Pr_0.3_Ba_2_Cu_3_O_6.67_	120	3.877	3.897	11.73

The thickness of all investigated films exceeds the critical thickness for strain relaxation in YBCO^54^. Note that the room-temperature *c*-axis lattice parameter of STO (*c* = 3.895 Å) is approximately one third of that of Y_0.7_Pr_0.3_Ba_2_Cu_3_O_7_. This makes it difficult to distinguish between substrate and film Bragg reflections.

### Gracing-incidence X-ray diffraction

High-energy grazing-incidence X-ray diffraction experiments were carried out at the P21.1 beamline^49^ at the PETRA III synchrotron (DESY - Hamburg). The setup is essentially identical to that described in ref. ^50^. The experimental results described here were obtained using a liquid nitrogen cryostat, allowing for a 100 K base temperature.

The data were collected using an incident angle *ϕ* = 0.030^∘^ from the sample surface, chosen to maximize the signal from Y_1−*x*_Pr_*x*_Ba_2_Cu_3_O_6+*y*_ films. The 3D dataset was then acquired by rotating the sample along the angle *ω* around the axis perpendicular to the beam axis in the range 30–230^∘^. The exposure time was 0.1 s per frame. X-ray energy was set to 101.769 keV. The 1D profiles shown in Fig. 1 were obtained by integrating the 3D datasets in *K* and *L* intervals of size ±0.05 and ±0.1 r.l.u., respectively.

### Resonant X-ray scattering (REXS)

REXS measurements were performed at the UE46-PGM1 beam line^51^ at the BESSY II electron storage ring. A helium cryostat permitted a base temperature of 10 K. *H*-*L* momentum maps were acquired by rocking the incident angle *θ* at different scattering *θ*_sc_ angles. The *H*, *L* values are then reconstructed for each point using the UB matrix, obtained by aligning the (0, 0, 2) and (1, 0, 3) Bragg reflections. X-ray absorption spectroscopy (XAS) was performed in electron-yield mode and allowed us to identify the Pr-*M*_5_ and Cu-*L*_3_ resonances. The *Q*_1_ and *Q*_2_ (CDW) peaks were extracted by fitting the spectra with a Gaussian plus a quadratic and linear background, respectively. All the spectra were normalized by the intensity of the incoming beam.

## Supplementary information

Supplementary Information

Transparent Peer Review file

## Data Availability

The data generated in this study and plotted in the figures in the main text and Supplementary Material have been deposited in the Zenodo database available at 10.5281/zenodo.18816715 (ref. ^53^). All figure data are saved in ASCII format and can be accessed using standard text editors.
