# Supplementary material for: Differentiation of site-specific symmetry breaking orders in Y1−xPrxBa2Cu3O6+y
Source: Nat Commun. 2026 Apr 29;17:5837. doi: 10.1038/s41467-026-72446-0 (PMC13332147; doi:10.1038/s41467-026-72446-0)
Supplement: Supplementary file 1 — Supplementary Information [file 41467_2026_72446_MOESM1_ESM.pdf]

Differentiation of Site-Sensitive Symmetry Breakings in $Y_{1-x}Pr_xBa_2Cu_3O_{7-\delta}$

S1. SAMPLE CHARACTERIZATION: LATTICE PARAMETERS

TABLE I. **Studied film and crystal systems.** Thicknesses (d) and room temperature lattice parameters of the three film systems used for this study. Films are relaxed from the $SrTiO_3$ (001)-substrate.

Compound	Type	d [nm]	a [Å]	b [Å]	c [Å]	$\epsilon = (b-a)/a$	Order	Ref.
$PrBa_2Cu_3O_7$	Film	90	3.895	3.895	11.82	≈ 0	$(\sim 1/3, 0, 0)$	This work
$PrBa_2Cu_3O_7$	Crystal	-	3.87	3.96	11.75	0.02	Unknown	1
$Y_{0.7}Pr_{0.3}Ba_2Cu_3O_7$	Film	60	3.858	3.907	11.65	0.0127	No order	This work
$Y_{0.7}Pr_{0.3}Ba_2Cu_3O_7$	Crystal	-	3.87	3.87	11.67	0	(∼1/3,0,1)	2
$Y_{0.7}Pr_{0.3}Ba_2Cu_3O_{6.67}$	Film	120	3.877	3.897	11.73	0.005	$(\sim 1/3, 0, [0, 1/2])$	This work
$Y_{0.775}Pr_{0.3}Ba_2Cu_3O_{6.6}$	Film	40	-	-	-	-	(∼1/3,0,1/2)	3
$YBa_2Cu_3O_{6.67}$	Crystal	-	3.81	3.87	-	0.016	Ortho-8, (∼ 1/3,0,1/2)	4

SAMPLES CHARACTERIZATION: RESISTIVITY

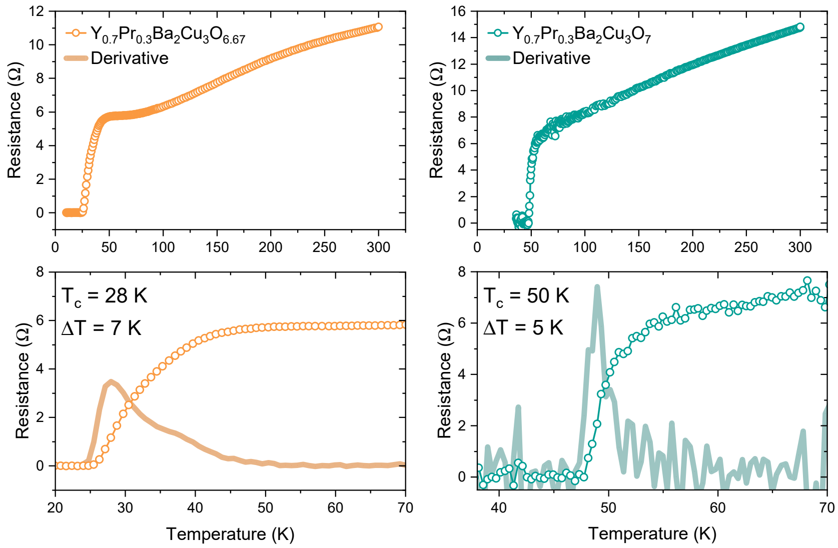

FIG. S1. **Resistivity of $Y_{1-x}Pr_xBa_2Cu_3O_{7-y}$ samples.** Resistance versus temperature measurements of $Y_{0.7}Pr_{0.3}Ba_2Cu_3O_{6.67}$ and $Y_{0.7}Pr_{0.3}Ba_2Cu_3O_7$ films. (Top panels) Resistance across the superconducting transition and up to room temperature. (Bottom panels) Zoom on the superconducting transition.

S2. EXPERIMENTAL RESOLUTION

We have estimated the experimental resolution for both GI-XRD and REXS measurements. In case of GI-XRD, the effective resolution depends both on experimental factors (monochromaticity, pixel size, divergence of beam) and on the reconstruction algorithm, which assigns a (H, K, L) set of coordinates to each pixel in the detector as a function of the rocking angle ω. In order to give an estimate of the combined effect of these two uncertainties, we have measured the Bragg reflections of the commercial $SrTiO_3$ substrates, which have high quality and cristallinity and are assumed to have an intrinsic broadening much smaller than the experimental resolution. By measuring 24 H-cuts at reflections with H,K,$L \leq 2$ (see Fig. S2), we determine $\Delta H = 0.015 \pm 0.004$ r.l.u. .

For REXS, the main contribution to the resolution comes from the finite size of the detector, an AXUV100-type photodiode (10 mm x 10 mm). Given the distance from the sample (30 cm) this gives an aperture of the scattering angle of ~ 1 deg, and a momentum resolution on H at the Q_1 reflection $(0.33, 0, 1)$ of 0.01 r.l.u.

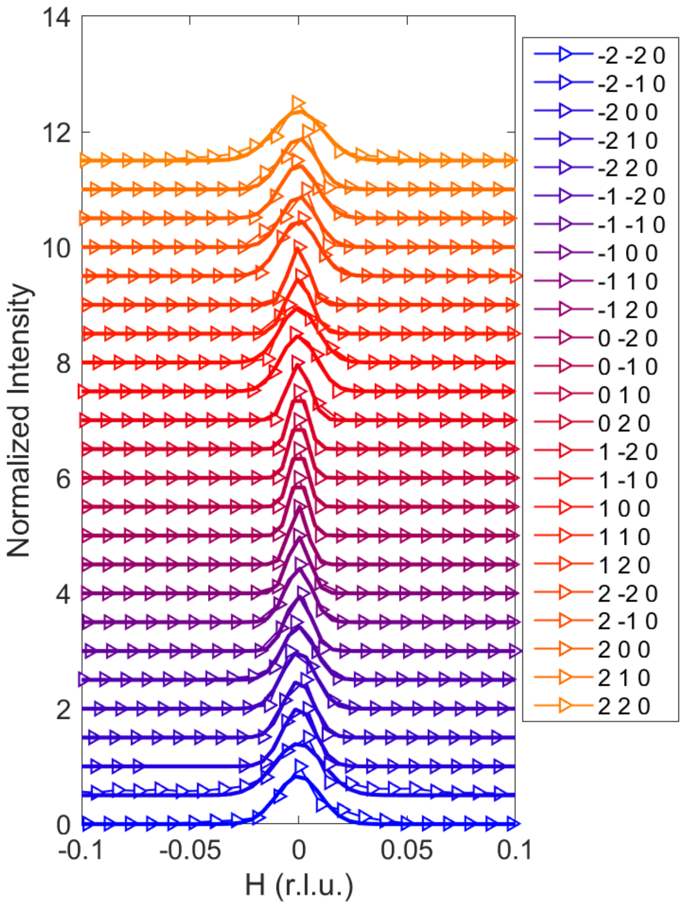

FIG. S2. **Bragg reflections in $SrTiO_3$.** Bragg reflections of $SrTiO_3$ used to determine experimental resolution for GI-XRD. Triangles are experimental H 1D cuts, obtained by integrating the 3D scattering volume in K and L over a ± 0.1 r.l.u. range. Solid lines represent Gaussian fits.

S3. L DEPENDENCE OF Q_1 AND Q_2

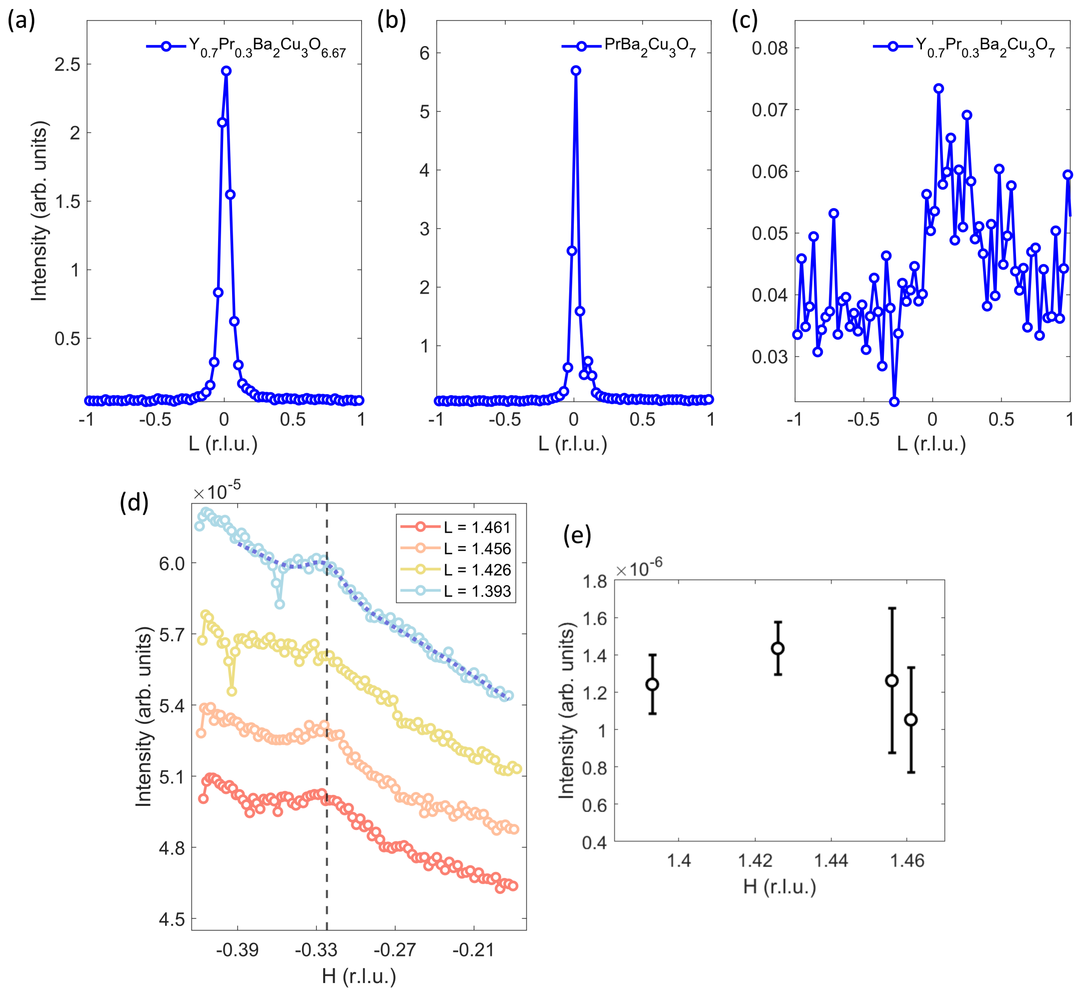

FIG. S3. ***L*-dependence of the Q_1 and Q_2 reflections.** (a-c): (-1,1,L) cut of the Q_1 superstructure peak in $Y_{0.7}Pr_{0.3}Ba_2Cu_3O_{6.67}$, $PrBa_2Cu_3O_7$, $Y_{0.7}Pr_{0.3}Ba_2Cu_3O_7$from left to right. (d) $(H, 0)$ dependence of the Q_2 peak measured in $Y_{0.7}Pr_{0.3}Ba_2Cu_3O_{6.67}$ by rocking the incident angle θ for different fixed scattering angles (168°, 164°, 156°, 148°). The legend labels refer to the L value (in r.l.u.) at the H corresponding to the peak. (e) Intensity of the Q_2 reflection estimated from a fitting model as describ d in the main text. Errorbars are 95% confidence intervals on fitting parameters.

S4. FITTING OF Q_1 ORDER IN SEVERAL BRILLOUIN ZONES

The use of hard x-ray diffraction allows us to estimate the width and modulation wavevector δ_1 (δ in the following for simplicity) using peaks in different Brillouin zones. In particular, we have fitted the Q_1 peaks using a Voigt function, with the width Gaussian component fixed to the extracted experimental resolution (see section above). To improve the quality of the fit, we have quantified δ_i as the distance between the superstructure peaks and the closest Bragg reflection (fitted with a Gaussian profile). *The uncertainty σ_i of the extracted parameter has been estimated as the σ-confidence interval (the reason for this choice is that we then use this error to compute a more precise uncertainty of the average wavevector.* The set of δ_i and their uncertainties is then used to quantify the average $\overline{\delta}$ and its uncertainty. A collection of the values obtained by fitting ∼30 1D-h cuts of the type $(H+h, K, L)$, with $H \leq 2$, $K = -1, 0, 1$ and $L = 0, 3$ is shown in Fig. S4. Evidently, the confidence intervals underestimate the spread of data. This might be due to an imperfect reconstruction algorithm. Therefore, we have decided to treat the extracted values as inconsistent datasets, and obtain a better estimate of the uncertainty using the so-called Brige ratio. In particular, assuming that the δ_i^1 are normal distributed with $\delta_i^1 \sim \mathcal{N}(\mu, \sigma^2)$, the standard way to extract $\overline{\delta}$ and $\overline{\Delta\delta_R}$ is:

$$\overline{\delta} = \frac{\sum_i w_i \cdot \delta_i}{\sum_i w_i}, \qquad \overline{\Delta\delta} = \frac{1}{\sqrt{\sum_i w_i}} \qquad \text{with} \quad w_i = \frac{1}{\sigma_i^2} \tag{S1}$$

In case of n inconsistent measurements, $\Delta\delta_R$ is simply multiplied by the Birge ratio[5]:

$$R = \sqrt{\frac{\chi^2}{\text{n-1}}} \qquad \text{with} \quad \chi^2 = \sum_i w_i \cdot (\delta_i - \overline{\delta})^2 \tag{S2}$$

We report $\overline{\delta}$ and the corrected uncertainty $\overline{\Delta\delta}_R$ as the red line and shading in Fig. S4. The values are:

Sample	Temperature	$\delta \pm \Delta\delta_R$ (r.l.u.)
$PrBa_2Cu_3O_7$	100 K	0.333 ± 0.0024
$Y_{0.7}Pr_{0.3}Ba_2Cu_3O_{6.67}$	100 K	0.334 ± 0.0016
	150 K	0.336 ± 0.0018
	200 K	0.334 ± 0.0022
	300 K	0.334 ± 0.0018

TABLE II. Wavevector δ and its uncertainty $\Delta\delta_R$ of Q_1 superstructure for $PrBa_2Cu_3O_7$ and $Y_{0.7}Pr_{0.3}Ba_2Cu_3O_7$.

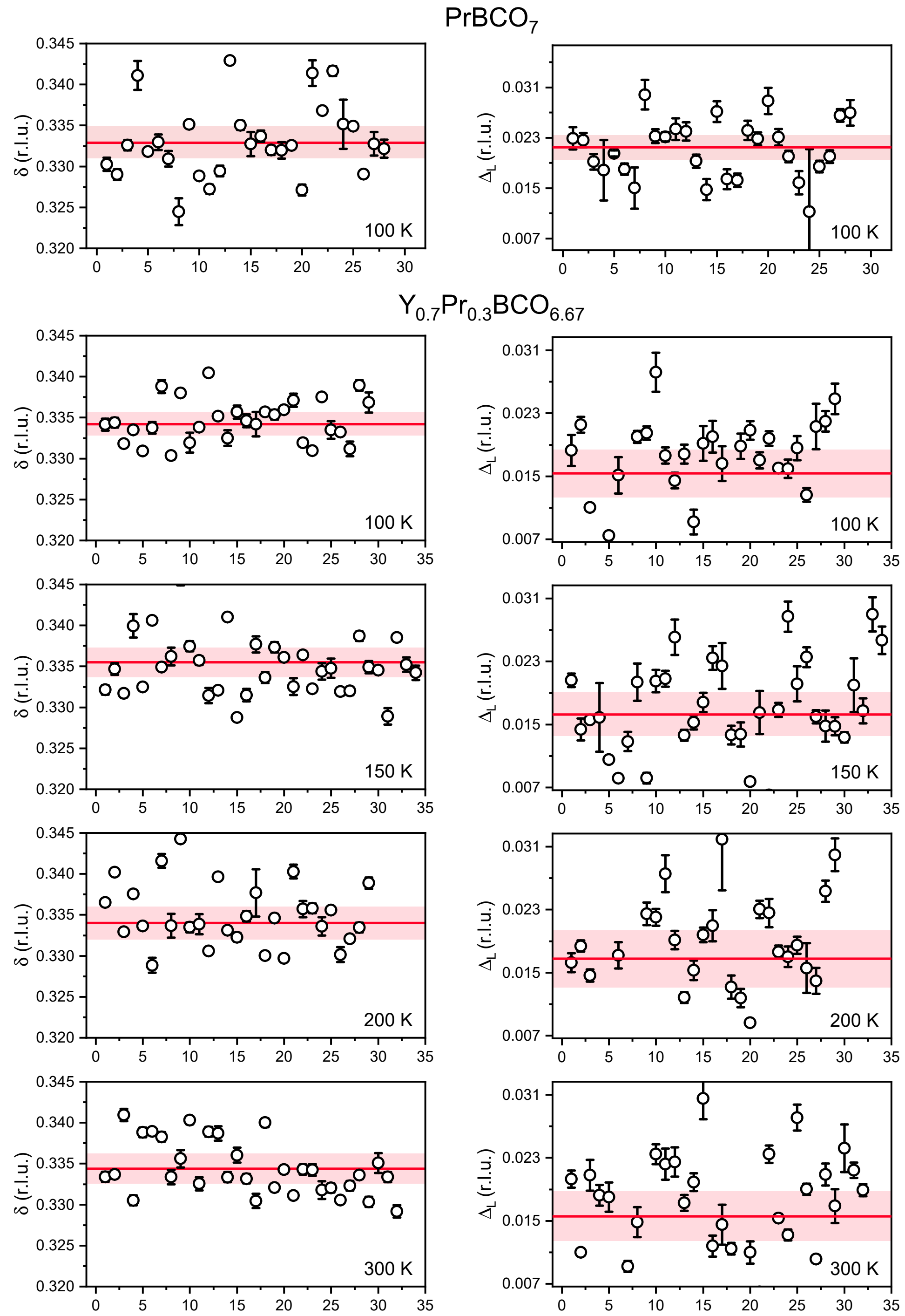

FIG. S4. **Estimation of δ and Δ in multiple zones.**. Spread of wavevector δ_1 and FWHM Δ_1 of Q_1 superstructure for $PrBa_2Cu_3O_7$ and $Y_{0.7}Pr_{0.3}Ba_2Cu_3O_7$. Temperature is reported in the bottom right part of the panels. The red solid line represents the weighted average of the datapoints, while the shadings highlight the regions defined by $[\overline{\delta} - \overline{\Delta\delta_R}, \overline{\delta} + \overline{\Delta\delta_R}]$. Errorbars on extracted δ and Δ_L are 95% confidence intervals of fitting parameters.

S5. STATISTICAL SIGNIFICANCE OF THE WEAK Q_2 ORDER PEAK

The fitting of the Q_2 reflection (Fig.2 in the main text) has been obtained using a model comprising a linear background and a Gaussian peak. The width and centre of the Gaussian peak have been fixed to the values obtained by fitting the spectrum at the Cu resonance. To verify that the addition of a non-zero peak is necessary to model the data, we proceeded in two ways. First, we have verified that the 2σ ($\sim 95\%$) confidence interval of the gaussian peak area remains strictly above zero. In particular, we obtain $A = (3.6 \pm 1.35) \cdot 10^{-5}$. Secondly, we have performed a p-value test on the null model (just a linear background, which properly fits the dataset at the Pr energy 930.95 eV, yellow label in Fig.2) and a model of a linear background with the addition of a Gaussian peak. We have assumed that the experimental points have an independent Gaussian noise $N(0, \sigma_N^2)$ and estimated $\sigma_N \sim 3 \cdot 10^{-6}$ by calculating the standard deviation in the part of the spectrum far from the Q_2 reflection (in the H range [0.20, 0.25] r.l.u.) after subtraction of the linear background. According to the Wilks theorem, the test statistics $-2\log(\Lambda) = 1/(\sigma_N^2)\sum(x_i - \mu)^2$ can be modelled by a $\chi^2(k)$ distribution under the null model, with k equal to the number of additional parameters. Therefore, the probability that the null model is correct is $p = 1 - \chi^2(-2\log(\Lambda), k)$ where $k = 1$ since we have fixed the peak center and width to the one obtained at the main Cu resonance, where the peak is evident (middle panel of Fig.3f). We obtain $p = 0.0042$ for the REXS scan mentioned by the Referee, and $p \sim 6 \cdot 10^{-12}$ for the scan at the Cu resonance (which instead has $k = 3$ additional free parameters compared to the null model since in this case we do not fix energy and width). Therefore, a peak is confidently present in the experimental spectra above Cu resonance. As a sanity check, taking the scan in the top of panel (f), where no peak is discernible, we obtain an amplitude of the peak of $0 \pm 0.015 \cdot 10^{-5}$ and a p-value of exactly 1 (not peak required with absolute certainty).

TABLE III. Summary of fit results at different photon energies.

Energy	Amplitude (arb. units)	p-value	K (free parameters)
930.95 eV	0 ± 0.0155	1	1
932.45 eV	0.102 ± 0.013	6×10^{-12}	3
933 eV	0.080 ± 0.029	0.0042	1

S6. FWHM OF Q_2 WITH TEMPERATURE

We report here the Full-Width-at-Half-maximum (FWHM) of the Q_2 (standard charge density wave) order, compared to the FWHM of $YBa_2Cu_3O_{6+x}$from Ref. 6.

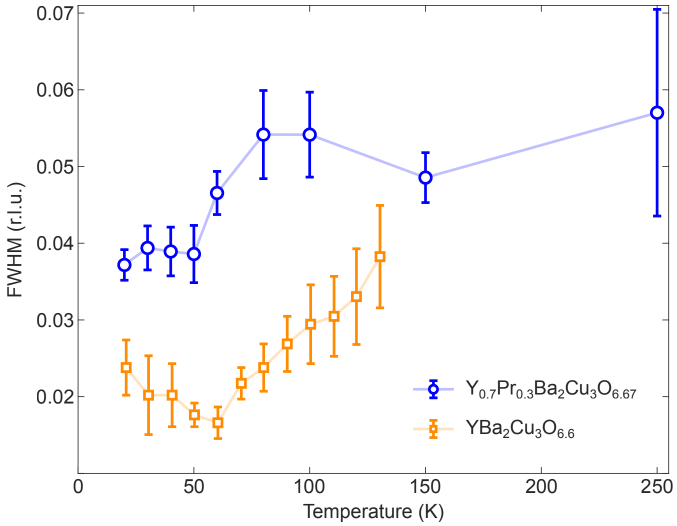

FIG. S5. **Comparison of FWHM of $Y_{0.7}Pr_{0.3}Ba_2Cu_3O_{6.67}$and $YBa_2Cu_3O_{6+x}$as a function of temperature.** Full-width-at-half-maximum of the Q_2 peak in $Y_{0.7}Pr_{0.3}Ba_2Cu_3O_{6.67}$ (blue dots) and $YBa_2Cu_3O_{6+x}$ (orange squares) (taken from Ref. 6) as a function of temperature. Errorbars are estimated as 95% confidence intervals on fitted parameters.

POSSIBLE SPACE GROUPS

TABLE IV. **Possible space groups.** Assuming either uniaxial ($Q = (1/3, 0, 0)$) or biaxial $Q = (1/3, 1/3, 0)$ order, this table is giving the possible space groups and associated number of Pr-modes. These space groups were extracted using the Isodistort software[7].

Ordering vector	$Q = (1/3, 0, 0)$		$Q = (1/3, 1/3, 0)$	
	Space group	Number of Pr-modes	Space group	Number of Pr-modes
	Pmmm, 47	1	*Pmmm*, 47	3
	Pmm2, 25	3	*Pmm2*, 25	4
			P2/m, 10	1
			Pm, 6	6

[1] E. Liarokapis, Condensed Matter **4**, 87 (2019), number: 4 Publisher: Multidisciplinary Digital Publishing Institute.
[2] A. Ruiz, B. Gunn, Y. Lu, K. Sasmal, C. M. Moir, R. Basak, H. Huang, J.-S. Lee, F. Rodolakis, T. J. Boyle, M. Walker, Y. He, S. Blanco-Canosa, E. H. da Silva Neto, M. B. Maple, and A. Frano, Nature Communications **13**, 6197 (2022).
[3] M. Kang, C. C. Zhang, E. Schierle, S. McCoy, J. Li, R. Sutarto, A. Suter, T. Prokscha, Z. Salman, E. Weschke, S. Cybart, J. Y. T. Wei, and R. Comin, Proceedings of the National Academy of Sciences **120**, e2302099120 (2023).
[4] J. Chang, E. Blackburn, A. T. Holmes, N. B. Christensen, J. Larsen, J. Mesot, R. Liang, D. A. Bonn, W. N. Hardy, A. Watenphul, M. v. Zimmermann, E. M. Forgan, and S. M. Hayden, Nature Physics **8**, 871 (2012).
[5] R. T. Birge, Physical Review **40**, 207 (1932).
[6] S. Blanco-Canosa, A. Frano, E. Schierle, J. Porras, T. Loew, M. Minola, M. Bluschke, E. Weschke, B. Keimer, and M. Le Tacon, Physical Review B **90**, 054513 (2014).
[7] B. J. Campbell, H. T. Stokes, D. E. Tanner, and D. M. Hatch, Journal of Applied Crystallography **39**, 607 (2006).
