# Supplementary material for: Differentiation of site-specific symmetry breaking orders in Y1−xPrxBa2Cu3O6+y
Source: Nat Commun. 2026 Apr 29;17:5837. doi: 10.1038/s41467-026-72446-0 (PMC13332147; doi:10.1038/s41467-026-72446-0)
Supplement: Supplementary file 2 — Transparent Peer Review file [file 41467_2026_72446_MOESM2_ESM.pdf]

nature portfolio

Peer Review File

Differentiation of Site-Specific Symmetry Breaking Orders in $Y_{1-x}Pr_xBa_2Cu_3O_{6+y}$

Corresponding Author: Dr Leonardo Martinelli

This file contains all reviewer reports in order by version, followed by all author rebuttals in order by version.

Version 0:

Reviewer comments:

Reviewer #1

(Remarks to the Author)
This study explores symmetry-breaking orders in the cuprate system Y1-xPrxBa2Cu3O6+y, with a particular focus on the distinct ordering phenomena that arise in this material. Using high-energy grazing incidence X-ray diffraction (GI-XRD) and resonant elastic X-ray scattering (REXS), the authors investigate structural and electronic orders in PrBa2Cu3O7 and Y0.7Pr0.3Ba2Cu3O6.67 films. Their main findings concern two distinct orders, labeled Q1 and Q2, which both exhibit similar in-plane periodicity (delta = ~1/3) but are associated with different mechanisms. Q1 resonates at Pr and out-of-plane copper sites, whereas Q2 resonates at in-plane copper sites. Q1 appears temperature independent and long ranged, suggesting a Pr-related superlattice structure. In contrast, Q2 is temperature dependent, vanishes at higher temperatures, and is interpreted as a charge-density wave (CDW) similar to that observed in YBCO. Based on measurements of three films—PrBa2Cu3O7, Y0.7Pr0.3Ba2Cu3O7, and Y0.7Pr0.3Ba2Cu3O6.67—together with previously published data, the authors construct a phase diagram to summarize their observations.
However, the key findings are not new. The Q1 order was already reported in Ref. 36 (Ruiz et al., Nat. Comm. 13, 6197 (2022)), where the reflection showed resonant enhancement at both the Pr M and Cu L edges, and its intensity persisted up to room temperature with only a slight decrease. Similarly, the Q2 order was described in Ref. 37 (Kang et al., PNAS 120, e2302099120 (2023)), which also reported a slightly different Q vector at the Pr M and Cu L edges. Although the present work notes minor variations, the overall results are highly similar to what has already been established. The authors also include data from Ref. 37 in their phase diagram by assigning an oxygen content of approximately y ≈ 0.8 for the studied sample, but they provide no justification for this value. Such ambiguity in oxygen stoichiometry, combined with the absence of clear reasoning, further weakens the conclusions. In summary, the results largely duplicate findings already available in the literature and do not convincingly advance understanding of symmetry-breaking orders in this system. The lack of clarity in interpreting discrepancies with previous studies further diminishes the impact of the work. For these reasons, I do not recommend publication of the paper in Nature Communications.
There are also several issues that require clarification. In Fig. 1(b), the non-resonant incommensurate peaks are not observed at L = 1, raising the question of whether these peaks are visible only under resonant conditions. If it is, can they be interpreted as superstructure peaks? In the same figure, the HK map of Y0.7Pr0.3Ba2Cu3O6.67 does not show the STO substrate peak that appears in PrBa2Cu3O7, and this discrepancy should be explained. In Fig. 2(b), the blue symbol and line graph should be explicitly labeled as Q2. The CDW or superstructure peaks also appear particularly weak and are not visible in Y0.7Pr0.3Ba2Cu3O7, whereas Ref. 36 clearly shows the CDW peak at the Pr M resonant edge in a single crystal of Y0.7Pr0.3Ba2O7. The authors need to address the origin of this discrepancy. Similarly, CDW peaks are not observed at L = 1.5 in PrBa2Cu3O7, in contrast to Ref. 37, which reported clear CDW peaks at both the Pr M and Cu L resonant edges. Again, the authors should clarify why their data diverge from earlier results.
Some definitions are also missing. The terms pZR and pFR, referring to the Zhang-Rice and Fehrenbacher-Rice states, should be explicitly defined on page 4. Finally, if the in-plane distortion of the Pr site associated with the ordering vector Q indeed gives rise to a superstructure, it should generate a reflection detectable even with soft X-ray diffraction. The absence of such a reflection is inconsistent with this interpretation and requires further discussion.

(Remarks on code availability)

Reviewer #2

(Remarks to the Author)
The Pr-doped $YBa_2Cu_3O_7$ (YBCO) system has attracted considerable interest following recent reports of two-dimensional (2D) (Ref. 36) and three-dimensional (3D) (Ref. 37) charge orders. However, these two studies present somewhat contradictory conclusions. A more comprehensive understanding of the charge orders in this system, particularly through resonant elastic and/or inelastic x-ray scattering (REXS or RIXS), is still lacking.

In this manuscript, L. Martinelli and coauthors employ grazing-incidence x-ray diffraction (GI-XRD) and REXS to investigate two distinct orders in $PrBa_2Cu_3O_7$ (PrBCO) and Pr-doped YBCO (PrYBCO) thin films. They demonstrate that the two orders exhibit nearly identical translational symmetry breaking. REXS reveals that one order corresponds to the previously reported charge order, while the other is related to a superlattice structure. This study offers valuable insights through a systematic investigation of charge orders in the Pr-doped YBCO system. The work is novel and significant as it demonstrates the coexistence of 2D and 3D orders and attributes them to different microscopic origins.

Overall, the reported results present significant advances in understanding charge orders in this system, although they also raise new questions. Given the novelty of the findings, I believe this work merits publication, provided the following ambiguities are addressed:

1. The authors state that Q_1 resonates at the Pr and out-of-plane Cu sites, whereas Q_2 resonates at the in-plane Cu site. It is unclear why the specific photon energies chosen in the energy-dependent scans (Fig. 2) are representative of in-plane versus out-of-plane Cu sites.

2. An L-dependent measurement could help confirm the 2D or 3D nature of the charge order. The authors only present L-dependence for Q1 in the SI. It is informative to show the L-dependence for Q2.

3. This work shows significant discrepancies compared with earlier reports (Refs. 36 & 37). The authors have addressed some of the differences in the discussion. Further discussion would be beneficial for the future direction of PrYBCO studies. For instance, Ref. 36 reports no 2D charge order at Q = (1/3, 0, 1.5) in PrYBCO, which contrasts strongly with the present results. Could this be due to differences between thin films and single crystals, or to variations in oxygen doping levels?

4. Similarly, this study finds no charge order in PrBCO, whereas Ref. 36 reports two in-plane charge orders. Please address this inconsistency.

5. The temperature-dependent measurements reveal contrasting behaviors for Q1 and Q2. It would be informative to know whether all the samples exhibit charge-density-wave–superconductivity competition below Tc for both Q1 and Q2.

(Remarks on code availability)

Reviewer #3

(Remarks to the Author)
In the manuscript titled "Differentiation of Site-Specific Symmetry Breaking Orders in $Y_{1-x}Pr_xBa_2Cu_3O_{6+y}$", Martinelli and co-authors report resonant x-ray scattering (REXS) and grazing-incidence diffraction (GI-XRD) measurements on epitaxial $PrBa_2Cu_3O_7$ and $Y_{1-x}Pr_xBa_2Cu_3O_{6+y}$ thin films grown on $SrTiO_3$ (STO) substrates. While $YBa_2Cu_3O_7$ (YBCO) is considered a model high-Tc cuprate in many aspects, Pr substitution uniquely suppresses superconductivity, vanishing entirely in $PrBa_2Cu_3O_7$, by most accounts. This suppression is frequently attributed to hole localization via hybridization between Pr-4f and O-2p orbitals [Ref. 6], though alternative explanations, including disorder effects [Mazin & Liechtenstein, Phys. Rev. B 57, 150 (1998)], have also been proposed.

The present work addresses this problem using REXS and GI-XRD. The authors identify two closely related ordering vectors: (a) a quasi-commensurate modulation at $Q2=(\delta 2,0,0.5)$, observed only in $Y_{0.7}Pr_{0.3}Ba_2Cu_3O_{6.67}$, which is strongest at the in-plane Cu-L resonance and exhibits temperature dependence consistent with a conventional CuO_2-plane charge density wave (CDW), and (b) a distinct modulation at $Q1 =(\delta 1,0,1)$, which is most intense at the Pr-M and out-of-plane Cu-L edges in $PrBa_2Cu_3O_7$ and $Y_{0.7}Pr_{0.3}Ba_2Cu_3O_{6.67}$, and out-of-plane Cu edges and shows little temperature dependence. Based on these resonance behaviors, the authors interpret Q1 as a Pr-related super-lattice structure, potentially connected to the suppression of superconductivity in $PrBa_2Cu_3O_7$.

The work presents a combination of REXS and GI-XRD to resolve multiple ordering phenomena in Pr-substituted cuprates. The experiments are technically challenging and demonstrates high experimental skill. The identification of Q1 and Q2 as distinct modulations with nearly identical in-plane periodicity but different resonance and temperature characteristics, is interesting and suggests new insights into site-specific electronic ordering. However, the results are limited to thin films on STO substrates, and without bulk-crystal data or cross-validation from complementary probes, it remains uncertain whether this order is intrinsic or influenced by strain, surface effects, or twinning. The work is potentially significant but presently falls short of providing unambiguous evidence for a distinct Pr-site symmetry-breaking order that directly competes with superconductivity. The main claims, in my opinion, are interesting but under-substantiated. I request that the authors address the following points prior to consideration for publication.

1. The conclusion that Q1 arises from Pr-sites is inferred primarily from resonance profiles. No polarization-resolved or azimuthal scattering analysis is provided, and self-absorption corrections are not discussed. Without tensor analysis of

scattering factors, the atomic specificity claim remains tentative. The authors should comment on this matter.

2. All measurements are on STO-grown, twinned thin films lacking chain order. Strain, surface fields, or twinning could stabilize or bias a super-lattice distinct from bulk behavior. No bulk crystals, thickness dependence, or alternative substrates are tested, leaving the generality of the observations unclear.

3. The claim that Q1 suppresses pairing is highly speculative. There is no systematic mapping across Tc, nor field-tuned data to reveal canonical CDW-SC competition. The superconducting implications are asserted more strongly than the presented data warrant. The authors should elaborate more on this aspect.

4. The authors state that the Q1 reflection is "resolution-limited" but do not provide deconvolved in-plane or out-of-plane correlation lengths or the instrumental resolution function. Providing this data would allow a quantitative assessment of the spatial extent of the order.

5. While possibly beyond the scope of the present manuscript, independent cross-validation through spatially-resolved techniques such as STM or STEM could really strengthen the evidence for a true Pr-related order. Their absence weakens confidence in the interpretation.

6. The peak shown in Fig. 2(f) at the out-of-plane Cu-L resonance for Y0.7Pr0.3Ba2Cu3O6.7 appears weak. Can the authors clarify whether it is statistically significant? The authors should also comment on possible effects of fluorescence background on the resonance profiles, particularly for weak reflections like Q2?

7. Have the authors quantified the uncertainty in δ1 and δ2 beyond the Gaussian fits? In Fig. 3(c), the scatter between non-resonant and resonant measurements suggests that the true uncertainty may be larger than the standard errors from the fits (for example, the 100 K non-resonant value differs noticeably from the resonant measurements). Since a small difference in the in-plane periodicity is used to distinguish Q1 and Q2, a more comprehensive statistical analysis that accounts for systematic uncertainties would strengthen the claim. Demonstrating reproducibility across multiple Brillouin zones or different experimental runs would further increase confidence. While I understand the time limitations for synchrotron experiments, I would urge the authors to consider this matter carefully.

8. Minor points:
There is a potential mislabeling in Fig. (b): Q1(blue) should be Q2?
The resistivity curve for Y0.7Pr0.3Ba2Cu3O7 seems noisy with some residual conductivity below Tc. While this is possibly due to a 2-probe measurement, please comment on it in text.
Ref. 6 in SI is improperly formatted.

(Remarks on code availability)
Not applicable.

Version 1:

Reviewer comments:

Reviewer #1

(Remarks to the Author)
The authors have provided a thorough and persuasive response to the concerns raised in the initial review. In particular, the primary critique regarding the novelty of the work has now been convincingly addressed. By clearly distinguishing the structural nature of the Q1 order from previously reported electronic modulations, the authors offer an important clarification and refinement of the existing literature.

I have one remaining point for consideration. Given that the observed ordering phenomena appear to be highly sensitive to film quality and growth conditions—such as strain and defect density—it would be useful to clarify to what extent the proposed phase diagram can be regarded as universal for the $Y_{1-x}Pr_xBa_2Cu_3O_{6+y}$ system rather than being specific to thin-film realization.

This point does not undermine the overall impact or significance of the study. I therefore recommend the manuscript for publication in Nature Communications.

(Remarks on code availability)

Reviewer #2

(Remarks to the Author)
As mentioned in my previous report, this work presents significant advances in understanding the physics of charge orders in the YPrBCO system. The authors have carefully and thoroughly addressed my comments as well as those of the other referees, and have revised both the manuscript and the Supplementary Materials accordingly.

The revised version provides additional and clearer insights into the phase diagram of Pr-doped YBCO, extending and clarifying previous studies (Refs. 36 and 37). I therefore conclude that the revised manuscript meets the standards for publication in Nature Communications, and I recommend acceptance as is.

(Remarks on code availability)

Reviewer #3

(Remarks to the Author)
I thank the authors for their careful and detailed responses and for the substantial revisions to the manuscript and Supplementary Material. Overall clarity, statistical analysis, and the balance of interpretation have improved significantly.

Most of my major concerns have been addressed. The discussion of site-specific resonances is now more appropriately nuanced, explicitly acknowledging possible overlap between out-of-plane Cu and Pr-related states. The expanded treatment of instrumental resolution, correlation lengths, and uncertainty estimation across multiple Brillouin zones strengthens the experimental basis of the conclusions.

Some limitations however remain. While the distinction between Q1 and Q2 is now better supported statistically, the weakest resonant features, particularly at the out-of-plane Cu-L resonance, remain close to the experimental sensitivity limit. Statistical analysis supports it's presence, but the significance depends on fixed peak parameters and background assumptions and should therefore be interpreted with appropriate caution. More generally, the microscopic origin of the Q1 superstructure is inferred indirectly from resonant behavior and scattering geometry. Independent real-space or site-resolved probes would provide a valuable cross-check, though this lies beyond the scope of the present work.

Overall, the revised manuscript presents a technically strong and conceptually clearer differentiation between two closely related ordering phenomena in Pr-substituted YBCO. With the more careful framing now adopted by the authors, I believe the work is suitable for publication.

(Remarks on code availability)

Open Access This Peer Review File is licensed under a Creative Commons Attribution 4.0 International License, which permits use, sharing, adaptation, distribution and reproduction in any medium or format, as long as you give appropriate credit to the original author(s) and the source, provide a link to the Creative Commons license, and indicate if changes were made.
In cases where reviewers are anonymous, credit should be given to 'Anonymous Referee' and the source.
The images or other third party material in this Peer Review File are included in the article’s Creative Commons license, unless indicated otherwise in a credit line to the material. If material is not included in the article’s Creative Commons license and your intended use is not permitted by statutory regulation or exceeds the permitted use, you will need to obtain permission directly from the copyright holder.

To view a copy of this license, visit https://creativecommons.org/licenses/by/4.0/

Reviewer #1 (Remarks to the Author):

This study explores symmetry-breaking orders in the cuprate system $Y_{1-x}Pr_xBa_2Cu_3O_{6+y}$, with a particular focus on the distinct ordering phenomena that arise in this material. Using high-energy grazing incidence X-ray diffraction (GI-XRD) and resonant elastic X-ray scattering (REXS), the authors investigate structural and electronic orders in $PrBa_2Cu_3O_7$ and $Y_{0.7}Pr_{0.3}Ba_2Cu_3O_{6.67}$ films. Their main findings concern two distinct orders, labeled Q1 and Q2, which both exhibit similar in-plane periodicity (delta = ~1/3) but are associated with different mechanisms. Q1 resonates at Pr and out-of-plane copper sites, whereas Q2 resonates at in-plane copper sites. Q1 appears temperature independent and long ranged, suggesting a Pr-related superlattice structure. In contrast, Q2 is temperature dependent, vanishes at higher temperatures, and is interpreted as a charge-density wave (CDW) similar to that observed in YBCO. Based on measurements of three films—$PrBa_2Cu_3O_7$, $Y_{0.7}Pr_{0.3}Ba_2Cu_3O_7$, and $Y_{0.7}Pr_{0.3}Ba_2Cu_3O_{6.67}$—together with previously published data, the authors construct a phase diagram to summarize their observations.

Authors: We thank the referee for the concise summary.

(1) However, the key findings are not new. The Q1 order was already reported in Ref. 36 (Ruiz et al., Nat. Comm. 13, 6197 (2022)), where the reflection showed resonant enhancement at both the Pr M and Cu L edges, and its intensity persisted up to room temperature with only a slight decrease. Similarly, the Q2 order was described in Ref. 37 (Kang et al., PNAS 120, e2302099120 (2023)), which also reported a slightly different Q vector at the Pr M and Cu L edges. Although the present work notes minor variations, the overall results are highly similar to what has already been established.

Authors: We respectfully disagree with the Referee comment, and we summarize our position below. The referee is right when arguing that there are some common points with the two scientific articles listed above, specifically the presence of 1) a peak at integer L position, reported in Ruiz et al, 2) a charge order peak (Q2 in our manuscript) at half-integer L-position, which was instead reported by Ref.37 but not Ref.36. However, our work however goes beyond Ref. 36 and 37 along several vectors. (1) The use of low-temperature, high-energy surface diffraction allows us to determine the nature of the Q1 reflection. While in Refs. 36 and 37 this was speculated to be a charge-order reflection (i.e. an electronic modulation), our measurements point instead towards a super-structural reflection involving Pr atoms. The low intensity of the (H,K)=(≈0.33,0) reflection reported in Ref.36 and 37, similar to a typical charge

order reflection, is demonstrated to be a consequence of an accidentally low structure factor in the first Brillouin zone (see Fig.R2 below). In other zones, the reflection shows a peak intensity 10^{-3} smaller than the strongest Bragg reflection, several order of magnitude bigger than charge order (for which this ratio is usually 10^{-6}). (2) Our study includes the stochiometric compound $PrBa_2Cu_3O_7$, with full oxygenation, while the authors in Ref. 36 report an oxygen content (from the wavevector of charge order, as we explain in point (2) below) of 6.8. (3) We exclude the presence of a doubled, purely Pr-related charge order peak in the stoichiometric $PrBa_2Cu_3O_{6+x}$ compound, reported in *Kang et al.* (Ref. 37). 4) We demonstrate that the Q_2 and Q_1 reflection sit at different L values **and** H values, an experimental fact that was not reported in Refs. 36-37. 5) We give an account of the possible distortion patterns related to the Pr atom given the *Pmmm* structure of the parent compound.

Overall, using data from two different, complementary x-ray techniques we reach new conclusions and we draw a more complete picture of the ordered phase in Pr-YBCO. We have revised the introduction section of the manuscript to highlight how our work extend significantly beyond the previous studies (Ref. 36 and 37).

"Here, we present a comprehensive study of PBCO and $Y_{1-x}Pr_xBa_2Cu_3O_{6+y}$ (x = 0.3, y = 0.67 and 1) relaxed films. Lattice and charge modulations are investigated by a combination of high-energy grazing incidence x-ray diffraction (GI-XRD) and resonant elastic x-ray scattering (REXS) (more precisely referred to as energy-integrated resonant inelastic x-ray scattering). Across the (x, y) phase diagram, we identify two ordering vectors: Q1 = (δ1, 0, 0), only present with Pr doping and absent in stoichiometric YBCO, and Q2 = (δ2, 0, 1/2) with δ1 ≈ δ2 ≈ 1/3. Although these two orders display similar in-plane modulation wavevectors, we demonstrate that they originate from different mechanisms and have diverging nature.
They resonate at different atomic sites. Q1 resonates at the Pr and out-of-plane copper sites, whereas Q2 resonates at the in-plane copper site. (2) They display different temperature dependences, as the Q2 order disappears above 250 K whereas the Q1 one is essentially temperature independent. (3) The two orders display different correlation lengths: Q1 is a long-range order with >100 Å, whereas Q2 has a much shorter correlation length. (4) The in-plane periodicity shows a minute discrepancy, in that we find $\delta_2 < 1/3 \lesssim \delta_1$.
We interpret the Q2 order in terms of a charge-density wave, identical to what has been reported in YBCO. Instead, based on the intensity of the peak over multiple zones, we provide strong evidence that the Q1 order is, in contrast to Refs. Ruiz et al. (2022) and Kang et al. (2023), a Pr-related superlattice structure. We demonstrate that the low intensity of the peak in the first Brillouin zone, which in Refs. Kang et al. (2023) and Ruiz et al. (2022) is comparable to the one of standard CDW, is due to an accidentally low structure factor. We argue that this additional superstructure must be incorporated to understand the unusual electronic properties of PBCO."

(2) The authors also include data from Ref. 37 in their phase diagram by assigning an oxygen content of approximately y ≈ 0.8 for the studied sample, but they provide no justification for this value. Such ambiguity in oxygen stoichiometry, combined with the absence of clear reasoning, further weakens the conclusions.

Editorial Note: Fig. R1 in this Peer Review File is adapted from M. Kang, C.C. Zhang, E. Schierle, S. McCoy, J. Li, R. Sutarto, A. Suter, T. Prokscha, Z. Salman, E. Weschke, S. Cybart, J.Y.T. Wei, & R. Comin, Discovery of charge orde r in a cuprate Mott insulator, Proc. Natl. Acad. Sci. U.S.A. 120 (30) e2302099120, https://doi.org/10.1073/pnas.2302 099120 (2023), licensed under a CC BY-NC-ND 4.0 license, https://creativecommons.org/licenses/by-nc-nd/4.0/

Authors: Our $Pr_xY_{1-x}Ba_2Cu_3O_{7-y}$ phase diagram spans Pr-for-Y substitution (*x*) on the vertical axis and oxygen content *y* on the horizontal axis. We have incorporated results from different sources, including Ref. 37 (Kang et al.) to draw a phase diagram for the presence of charge order (Q2) and superstructure (Q1) based on the current literature, including our work. **Fig. R1** displays **Fig. 3b** from the paper of *Kang et al.* (Ref. 37 in the initial version of this manuscript). A starting point for their study is x = 0, thus unsubstituted $YBa_2Cu_3O_{7-y}$ (YBCO). As can be seen in their figure, the authors report an oxygen deficiency for this sample of 0.2, (i.e. oxygen content *y*=0.8) based on the wavevector of the measured two-dimensional charge order. This estimate also agrees with the ≈90 K critical temperature reported by the authors (see e.g. [**R. liang et al., Phys. Rev. B 73, 180505(R) (2006)**]). The authors indeed calculate a doping $p < 0.15$ from the critical temperature (see table S1 in the Supplemental Material of Ref.37). As no precise oxygen content is reported in the manuscript for each sample, we assume that the oxygen content stays constant and Pr content *x* is varied (the authors only state that the oxygen deficiency is $\ll 1$). We have therefore plotted result from Kang et al., with a constant oxygen content of 6.8 in our phase diagram.

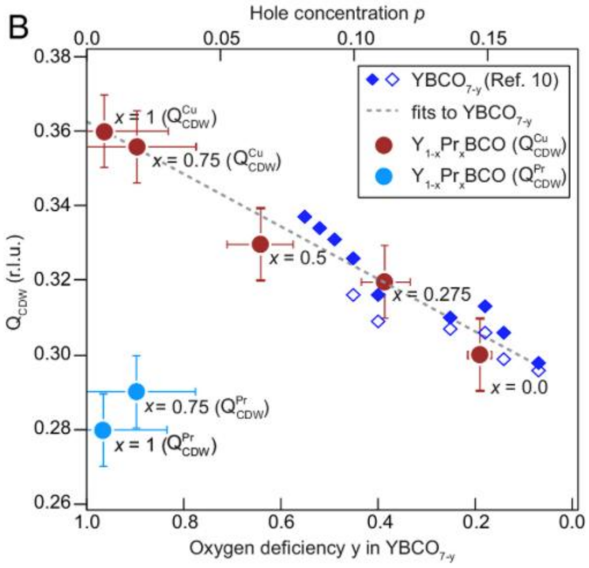

Fig. R1: Figure 3b from Kang et al. (Ref.37 in the initial version of manuscript).

(3) In summary, the results largely duplicate findings already available in the literature and do not convincingly advance understanding of symmetry-breaking orders in this system. The lack of clarity in interpreting discrepancies with previous studies further diminishes the impact of the work. For these reasons, I do not recommend publication of the paper in Nature Communications.

Authors: We respectfully disagree with the Referee's assessment that our results largely duplicate existing literature. As already detailed in our response to point (1), the presence of experimental reference points does not constitute duplication. Our work provides a much deeper discussion on the nature of reflection Q_1, thanks to the use of two different experimental techniques (one of them never employed before for these studies). On the basis of this expanded experimental dataset, we demonstrate that the Q_1 reflection originates from a structural distortion rather than from an electronic charge order, in clear contrast to earlier interpretations. We also discuss new conclusions as to why Pr-doped $Y_{1-x}Pr_xBa_2Cu_3O_{6+y}$ displays unusual electronic properties. These insights are not found in Ref. 36, 37 or other prior publications.

We also point out that the existing literature is not only scarce and recent, but also far from delivering a consistent picture of the observed phenomenology. Refs. 36 and 37 investigate samples with different oxygen and Pr concentrations, explore different out-of-plane momenta

(L=1 and L=1.5), and report qualitatively different experimental behaviour, for example regarding the temperature dependence or the presence of a double structure. In this context, our manuscript does not duplicate previous work, but instead provides a necessary and unifying development that clarifies and reconciles these earlier observations.

(4) There are also several issues that require clarification. In Fig. 1(b), the non-resonant incommensurate peaks are not observed at L = 1, raising the question of whether these peaks are visible only under resonant conditions. If it is, can they be interpreted as superstructure peaks?

Authors: We thank the Referee for pointing out this only apparent inconsistency. However, we stress that Fig.1c (in the revised manuscript) report the (H,L) plane at K=1, which is not the reflection explored by resonant scattering (which instead is performed at K=0). Below (**Fig. R2**), we report the H profile obtained integrating the (H,K,L) scattering volume in a window (with the same size as the ones used in Fig.1) centred around K=0 and L=1, which is the zone explored in resonant scattering measurements. Two peaks are visible at incommensurate values, corresponding to the Q_1 reflections (≈0.33,0,1) and (≈0.67,0,1). These signal-to-background ratio of these two peaks is only ~ 0.08, much smaller than the value of ~ 50 for the equivalent peak at K=1 and L=0 shown in Fig.1f (in revised manuscript). We speculate that this difference in intensity arises from the specific structure factor of the modulation. This explains why, in resonant scattering, the Q_1 and Q_2 reflections possess comparable intensity over the background, while the Q_1 is clearly stronger in other zones. It also explains why this peak could be confused with a charge-order reflection when only the (0.33,0,1) reflection is analysed (i.e. in soft x-ray resonant scattering experiments, like in *Ruiz et al*. (Ref.36)).

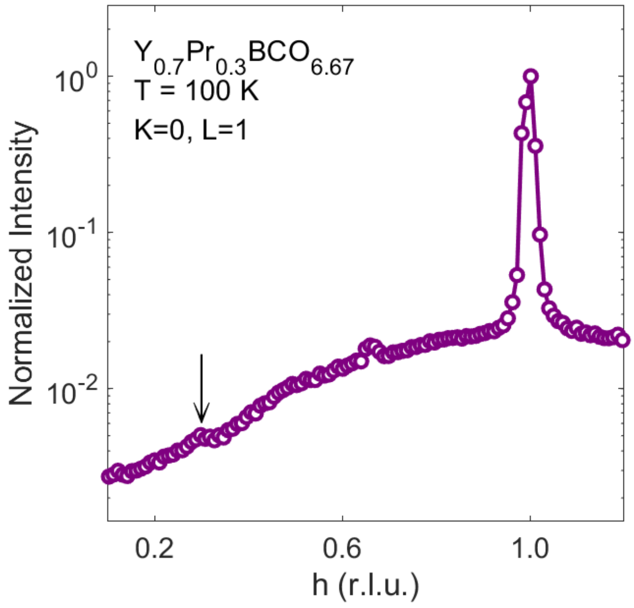

Fig. R2: H Profile at K=0 and L=1 r.l.u.. The Q_1-peak is visible at incommensurate positions close to H=0.33, as indicated by the black arrow.

(5) In the same figure, the HK map of Y0.7Pr0.3Ba2Cu3O6.67 does not show the STO substrate peak that appears in PrBa2Cu3O7, and this discrepancy should be explained.

Authors: The film and the substrate are having essentially the same in-plane lattice parameters but different *c*-axis lattice parameter. The substrate Bragg peaks are therefore best identified in the out-of-plane reciprocal direction (shown in panel c and d). At room temperature, the STO and $Y_{0.7}Pr_{0.3}Ba_2Cu_3O_{6.67}$ *c*-axis lattice parameters are respectively c_{STO}=3.895 Å and c_{YPrBCO}=11.65 Å. As 3xc_{STO}=11.685 Å$\approx c$, substrate and film Bragg peaks are very difficult to differentiate. We have added this information to in the method section of the revised manuscript.

(6) In Fig. 2(b), the blue symbol and line graph should be explicitly labelled as Q2.

Authors: We have corrected this labelling typo in Fig. 2b and we have added Q1 and Q2 at the top of the panels to help the reader.

(7) The CDW or superstructure peaks also appear particularly weak and are not visible in Y0.7Pr0.3Ba2Cu3O7, whereas Ref. 36 clearly shows the CDW peak at the Pr-M5 resonant edge in a single crystal of Y0.7Pr0.3Ba2O7. The authors need to address the origin of this discrepancy.

Authors: We thank the Referee for pointing out this discrepancy with respect to Ref.36 (Ruiz et al.). Indeed, Ref. 36 mentions the absence of 2D charge order (L=half integer) with a reference to their supplementary information, in agreement with our observation in $Pr_{0.3}Y_{0.7}Ba_2Cu_3O_7$. The difference with Ref. 36 is therefore only limited to the different intensity of the structural peak, being the Q1 reflection in our $Pr_{0.3}Y_{0.7}Ba_2Cu_3O_7$ sample much weaker than in single crystals shown in Ref. [36] (but not totally absent, as shown in Fig.S3 in our Supplemental Material). We attribute this difference primarily to the reduced structural coherence of the Pr-induced modulation in thin films, where epitaxial strain, higher defect density and finite thickness are known to weaken long-range ordering of subtle structural modulations (as observed, for instance, for the CuO-chain order in YBCO films). An additional contribution may arise from small but systematic deviations in the actual Pr content of thin films relative to the nominal composition. In PLD growth, different sticking coefficients and the higher volatility of Pr compared to Y can lead to an effective substitution level on the Y site that slightly deviates from the target stoichiometry.

We have now significantly revised the Discussion section to include these changes:

"This discrepancy might be related to the reduced structural coherence of the Pr-induced modulation in thin films, where epitaxial strain, higher defect density, and finite thickness are known to weaken long-range ordering of subtle structural modulations (as observed, for instance, for the CuO-chain order in YBCO films). An additional contribution may arise from small deviations in the actual Pr content of thin films relative to the nominal composition. In Pulsed Laser Deposition growth, different sticking coefficients and the higher volatility of Pr compared to Y can lead to an effective substitution level on the Y site that slightly deviates from the target stoichiometry.

Ref.36, on the other hand, reports the presence of two Pr- and Cu-resonating peaks at half-integer L = 1.5 in $PrBCO_7$, and a single weak CDW peak in $Y_{1-x}Pr_xBa_2Cu_3O_7$ (although the authors estimate a small oxygen deficiency ~0.2). This last result is in agreement with our Q_2 order. However, neither this work nor Ref.37 finds the presence of any Pr-related or Cu-related orders in PBCO at L = 1.5. We can speculate that the small oxygen deficiency y ~ 6.8 of the films used in the work may play a role, as well as the smaller thickness of the films employed (40 nm). It would also be of great interest to investigate whether the samples used in Ref.36 show the presence of any structure at integer L positions."

(8) Similarly, CDW peaks are not observed at L = 1.5 in PrBa2Cu3O7, in contrast to Ref. 37, which reported clear CDW peaks at both the Pr M and Cu L resonant edges. Again, the authors should clarify why their data diverge from earlier results.

Authors: We thank the author for pointing out the difference between our results and Ref.37 (Kang et al.). We speculate that the discrepancy might be due to a different oxygen content, whose value has a strong influence on the superstructure, as we demonstrate with our results. As explained in point (2), we infer that the films used in Ref.37 have an oxygen content closer to 6.8 rather than 7 (based on the wavevector of charge order and the estimated doping of 0.147 provided by the authors in Table S1). Additionally, we point out that: 1) the authors do not report scans across L=1 or L-dependence of the measured peaks, so that we are unable to verify whether the Pr-related superstructure is visible in their samples; 2) the standard charge order at half-integer L (L=1.5), observed in Ref.37, is also not visible in Ref.36 for the same Pr concentration of ~ 0.3.

Some definitions are also missing. The terms pZR and pFR, referring to the Zhang-Rice and Fehrenbacher-Rice states, should be explicitly defined on page 4.

Authors: We thank the referee for this comment and in the revised text, pZR and PFR is now explicitly defined. The text now reads:

"Generally, we label p_{ZR} for the hole concentration within the CuO_2 planes. By contrast, holes hybridizing between oxygen and Pr sites are labelled with p_{FR}. In this fashion, we consider both Zhang-Rice and Fehrenbacher-Rice configurations. The sum $p_{ZR}+p_{FR}$ is bounded by the total hole doping p - controlled by the oxygen content."

Finally, if the in-plane distortion of the Pr site associated with the ordering vector Q indeed gives rise to a superstructure, it should generate a reflection detectable even with soft X-ray diffraction. The absence of such a reflection is inconsistent with this interpretation and requires further discussion.

Authors: We observe the superstructure peak with both resonant (Fig. 2c) and non-resonant x-ray scattering (Fig. 1). The fact that the superstructure seems weak with soft x-rays, i.e. with an intensity only one order of magnitude larger than the charge order reflection, stems from a low structure factor value of the (0.33, 0, 1) reflection (see again Fig. R2).

Reviewer #2 (Remarks to the Author):

The Pr-doped $YBa_2Cu_3O_7$ (YBCO) system has attracted considerable interest following recent reports of two-dimensional (2D) (Ref. 36) and three-dimensional (3D) (Ref. 37) charge orders. However, these two studies present somewhat contradictory conclusions. A more comprehensive understanding of the charge orders in this system, particularly through resonant elastic and/or inelastic x-ray scattering (REXS or RIXS), is still lacking.

In this manuscript, L. Martinelli and coauthors employ grazing-incidence x-ray diffraction (GI-XRD) and REXS to investigate two distinct orders in $PrBa_2Cu_3O_7$ (PrBCO) and Pr-doped YBCO (PrYBCO) thin films. They demonstrate that the two orders exhibit nearly identical translational symmetry breaking. REXS reveals that one order corresponds to the previously reported charge order, while the other is related to a superlattice structure. This study offers valuable insights through a systematic investigation of charge orders in the Pr-doped YBCO system. The work is novel and significant as it demonstrates the coexistence of 2D and 3D orders and attributes them to different microscopic origins.

Overall, the reported results present significant advances in understanding charge orders in this system, although they also raise new questions. Given the novelty of the findings, I believe this work merits publication, provided the following ambiguities are addressed:

Authors: We thank the referee for acknowledging the need for this study and for the positive recommendation. Below we address the raised points, also mentioning how we have improved the manuscript accordingly.

1. The authors state that Q_1 resonates at the Pr and out-of-plane Cu sites, whereas Q_2 resonates at the in-plane Cu site. It is unclear why the specific photon energies chosen in the energy-dependent scans (Fig. 2) are representative of in-plane versus out-of-plane Cu sites.

Authors: We thank the Referee for this comment, which stimulated us to improve the discussion on the matter. In assigning the higher-energy tail of the XAS to out-of-plane (i.e. chain) Cu states we were relying on previous REXS work (Phys. Rev. Lett. **109**, 167001 (2012)) on YBCO where the in- and out-of-plane Cu resonances were established. In this mentioned work, it is demonstrated that the out-of-plane (chain) Cu resonance occurs at a higher energy compared to the in-plane Cu resonance. However, by analysing the published work on XAS of $Y_{1-x}Pr_xBa_2Cu_3O_{6+y}$, we realised that spectral weight above the main, in-plane Cu resonance could also be due to Pr-related states. Indeed, e.g. from Ref. 36 in the previous version of the manuscript (Kang et al.), a monotonic increase in the spectral weight upon Pr-substitution is visible (Fig. 1D). A possible interpretation is the presence of Pr^{4+} states, that sit at a higher energy than the main Pr^{3+}, and whose presence is predicted by the most accepted theoretical model for the electronic structure of PBCO [Fehrenbacher, R., & Rice, T. M. PRL, ***70***, 3471–3474 (1993)]. We have therefore chosen to propose both interpretations in the manuscript. Specifically, we state:

"*The in-plane and out-of-plane Cu resonances are split due to different crystal-field environment - consistent with previous work on YBCO (PRL **109**, 167001 (2012). Our XAS data does not exclude the existence of a weaker Pr^{4+} resonance overlapping with the out-of-plane Cu resonance.*"

2. An L-dependent measurement could help confirm the 2D or 3D nature of the charge order. The authors only present L-dependence for Q1 in the SI. It is informative to show the L-dependence for Q2.

Authors: We thank the Referee for pointing out that the L-dependence of charge order was not adequately shown in the manuscript. We have now improved Fig. S3 and S4 in the Supplementary Material (new Fig.S2) to highlight the difference in the L-dependence of Q1 and Q2. The measured trend is consistent with previous report for charge order in other cuprates, with a mild L-dependence.

3. This work shows significant discrepancies compared with earlier reports (Refs. 36 & 37). The authors have addressed some of the differences in the discussion. Further discussion would be beneficial for the future direction of PrYBCO studies. For instance, Ref. 36 reports no 2D charge order at Q = (1/3, 0, 1.5) in PrYBCO, which contrasts strongly with the present results. Could this be due to differences between thin films and single crystals, or to variations in oxygen doping levels?

Authors We thank the Referee for this comment. Indeed, reference 36 (Ruiz et al.) mentions the absence of 2D charge order (L=half integer) with a reference to their supplementary information, in agreement with our observation in $Pr_{0.3}Y_{0.7}Ba_2Cu_3O_7$. The difference with Ref. 36 is therefore only limited to the different intensity of the structural peak, being the Q1 reflection in our $Pr_{0.3}Y_{0.7}Ba_2Cu_3O_7$ sample much weaker than in single crystals shown in Ref. [36]. We attribute this difference primarily to the reduced structural coherence of the Pr-induced modulation in thin films, where epitaxial strain, higher defect density and finite thickness are known to weaken long-range ordering of subtle structural modulations (as observed, for instance, for the CuO-chain order in YBCO films). An additional contribution, potentially the key factor underlying the apparent discrepancy between crystals and films, may arise from small but systematic deviations in the actual Pr content of thin films relative to the nominal composition. In PLD growth, different sticking coefficients and the higher volatility of Pr compared to Y can lead to an effective substitution level on the Y site that slightly deviates from the target stoichiometry. Even a tiny reduction in the actual Pr concentration can substantially diminish the amplitude of the Pr-induced lattice modulation, and hence the observable superlattice peak intensity.

When comparing our work with Ref. 37 (Kang *et al.*), one should note that the oxygen concentrations are different. The work involves films that, according to the estimation given by authors, have a sizeable oxygen deficiency of $y \sim 0.2$ (see Fig. R1). To make this more transparent to the reader, we have we now state directly in the caption of Fig. 3 of the main text that oxygen concentrations are directly extracted from literature.

In conclusion, we believe that the differences from the existing literature stem from: (i) the reduced structural coherence and slight deviations in the effective Pr content that can occur in thin films, in comparison with Ref. 36; (ii) the different oxygen concentration, which according to our study has a strong impact on the presence of electronic/structural orders, in comparison with Ref. 37.

4. Similarly, this study finds no charge order in PrBCO, whereas Ref. 36 reports two in-plane charge orders. Please address this inconsistency.

Authors: We assume that the referee is referring to the work of Kang et al. (Ref. 37). In the work of Kang et al., the authors report the presence of an additional, temperature-independent peak for high substitution of Pr ($Y_{0.2}Pr_{0.8}BCO_y$ and $PrBCO_y$). We speculate two reasons to account for the different phenomenology. First, the authors in Ref.37 estimated an oxygen concentration of 6.8 (see reply to referee 1, point 2) whereas our films are fully oxygenated. An oxygen content closer to the "underdoped" regime in YBCO might be favourable to standard (L=1.5) charge order and might facilitate its detection. Indeed, we clearly find the presence of a Q2 order in our oxygen-depleted sample. Secondly, the thickness of the films used in Ref. 37 is smaller than ours (40 nm vs. 90 nm), so that effects from the substrates (e.g. strain) might be more relevant. Given these differences, our results are not necessarily inconsistent with Ref.37.
We report, moreover, that the results of Ref. 37 are also not fully consistent with Ref. 36, in that no peak at L=1.5 is reported in Ref. 36 for moderate Pr substitution (x=0.3).

To make this point clearer, we have revised the text defining the phase diagram that include our results and those from Ref. 36 and 37. We have also extended the discussion section by adding a paragraph to discuss the relationship between our results and the existing literature.

"This discrepancy might be related to the reduced structural coherence of the Pr-induced modulation in thin films, where epitaxial strain, higher defect density, and finite thickness are known to weaken long-range ordering of subtle structural modulations (as observed, for instance, for the CuO-chain order in YBCO films). An additional contribution may arise from small deviations in the actual Pr content of thin films relative to the nominal composition. In Pulsed Laser Deposition growth, different sticking coefficients and the higher volatility of Pr compared to Y can lead to an effective substitution level on the Y site that slightly deviates from the target stoichiometry.
Ref.36, on the other hand, reports the presence of two Pr- and Cu-resonating peaks at half-integer L = 1.5 in $PrBCO_7$, and a single weak CDW peak in $Y_{1-x}Pr_xBa_2Cu_3O_7$ (although the authors estimate a small oxygen deficiency ~0.2). This last result is in agreement with our Q_2 order. However, neither this work nor Ref.37 finds the presence of any Pr-related or Cu-related orders in PBCO at L = 1.5. We can speculate that the small oxygen deficiency y ~ 6.8 of the films used in the work may play a role, as well as the smaller thickness of the films employed (40 nm). It would also be of great interest to investigate whether the samples used in Ref.36 show the presence of any structure at integer L positions."

5. The temperature-dependent measurements reveal contrasting behaviors for Q1 and Q2. It would be informative to know whether all the samples exhibit charge-density-wave–superconductivity competition below Tc for both Q1 and Q2.

Authors: We thank the author for this remark. Indeed, demonstrating a correlation between Q2 and superconductivity over the phase diagram of $Y_{1-x}Pr_xBa_2Cu_3O_{7-y}$ would draw an important conclusion over the competition between charge order and superconductivity. However, in this manuscript, we have studied three films of $Pr_xY_{1-x}Ba_2Cu_3O_{7-x}$: (x,y) = (1,0),(0.3,0) and (0.3,0.33).

Only the last-mentioned film display both Q_1 and Q_2 order. In the other two samples, no trace of Q_2 is found. This is the reason why temperature dependence of both orders is compared only for this sample. We will leave a more general study, involving different combination of Pr and oxygen doping, for future studies.

Reviewer #3 (Remarks to the Author):

In the manuscript titled "Differentiation of Site-Specific Symmetry Breaking Orders in Y1−xPrxBa2Cu3O6+y", Martinelli and co-authors report resonant x-ray scattering (REXS) and grazing-incidence diffraction (GI-XRD) measurements on epitaxial PrBa2Cu3O7 and Y1−xPrxBa2Cu3O6+y thin films grown on SrTiO3 (STO) substrates. While YBa2Cu3O7 (YBCO) is considered a model high-Tc cuprate in many aspects, Pr substitution uniquely suppresses superconductivity, vanishing entirely in PrBa2Cu3O7, by most accounts. This suppression is frequently attributed to hole localization via hybridization between Pr-4f and O-2p orbitals [Ref. 6], though alternative explanations, including disorder effects [**Mazin & Liechtenstein, Phys. Rev. B 57, 150 (1998)**], have also been proposed.

The present work addresses this problem using REXS and GI-XRD. The authors identify two closely related ordering vectors: (a) a quasi-commensurate modulation at Q2=(δ2,0,0.5), observed only in Y0.7Pr0.3Ba2Cu3O6.67, which is strongest at the in-plane Cu-L resonance and exhibits temperature dependence consistent with a conventional CuO2-plane charge density wave (CDW), and (b) a distinct modulation at Q1 =(δ1,0,1), which is most intense at the Pr-M and out-of-plane Cu-L edges in PrBa2Cu3O7 and Y0.7Pr0.3Ba2Cu3O6.67, and out-of-plane Cu edges and shows little temperature dependence. Based on these resonance behaviors, the authors interpret Q1 as a Pr-related super-lattice structure, potentially connected to the suppression of superconductivity in PrBa2Cu3O7.

The work presents a combination of REXS and GI-XRD to resolve multiple ordering phenomena in Pr-substituted cuprates. The experiments are technically challenging and demonstrates high experimental skill. The identification of Q1 and Q2 as distinct modulations with nearly identical in-plane periodicity but different resonance and temperature characteristics, is interesting and suggests new insights into site-specific electronic ordering. However, the results are limited to thin films on STO substrates, and without bulk-crystal data or cross-validation from complementary probes, it remains uncertain whether this order is intrinsic or influenced by strain, surface effects, or twinning. The work is potentially significant but presently falls short of providing unambiguous evidence for a distinct Pr-site symmetry-breaking order that directly competes with superconductivity. The main claims, in my opinion, are interesting but under-substantiated. I request that the authors address the following points prior to consideration for publication.

Authors: We thank the referee for the concise summary and for recognizing the experimental challenges associated with the RIXS and GI-XRD experiments. Their constructive comments have greatly helped us in improving the manuscript. We provide below an answer to every point raised by the Referee, also mentioning how the manuscript has been changed accordingly.

1. The conclusion that Q1 arises from Pr-sites is inferred primarily from resonance profiles. No polarization-resolved or azimuthal scattering analysis is provided, and self-absorption corrections are not discussed. Without tensor analysis of scattering factors, the atomic specificity claim remains tentative. The authors should comment on this matter.

Authors: To establish the Pr^{3+}-resonance energy, we used different substitutions of Pr and Y. This is shown in Figure 2. When the Pr concentration is reduced be a factor 0.3, the absorption

resonance reduces by the same factor while the copper L-resonances remains approximately constant. Additionally, the shape of the Pr3+ resonance is known from previous experiments on Pr-oxides (see e.g. [**J. Herrero-Martín, et al., *Phys. Rev. B* 84, 115131 (2011)**] and consists of a single main peak. We also note that our Pr^{3+} and in-plane Cu resonance labelling is consistent with previous literature (Kang et al. PNAS 2023). Therefore, we argue that the intense peak on the left side of the Cu^{2+} resonance can be assigned to Pr with a high degree of certainty. We emphasise this in the revised manuscript by stating:

"*The labeling of these two close-lying resonances is reached by varying the Pr concentration. The first peak in the XAS resonance clearly scales with Pr content — see Fig. 2a and Fig. 2d — while the second feature at 930.95 eV shows less dependence on the Pr content. The Pr resonance is likely complex and composed of multiple peaks. Previous XANES experiments (Merz et al., 1997) suggested a Pr valence of approximately 3.2, so that a 20% contribution of Pr^{4+}states is expected. The Pr^{4+}contribution peaks around 1 eV higher than $Pr^{3+}$and has a broad lineshape (see Herrero-Martín et al., 2011), so that it partially overlaps with the Cu white line*."

We also thank the Referee for proposing to consider self-absorption corrections. We verified that the magnitude of the self-absorption correction as a function of momentum (i.e. as a function of incident angle) is small (<20%). This is because the Q1 order is reached in a very grazing-incidence condition ($\theta < 15°$) at 930 eV. A plot of the self-absorption correction is given below (**Fig. R3**) using an extended version of the formula given e.g. in [**R. Fumagalli et al., *Phys. Rev. B* 99, 134517 (2019)]** adapted for thin films, for zero energy loss and scattering angle of 108° (used to intercept the maximum of the Q1 peak at $L = 1$). We have now replaced the plots in Fig.2e-f with the version corrected by self-absorption.

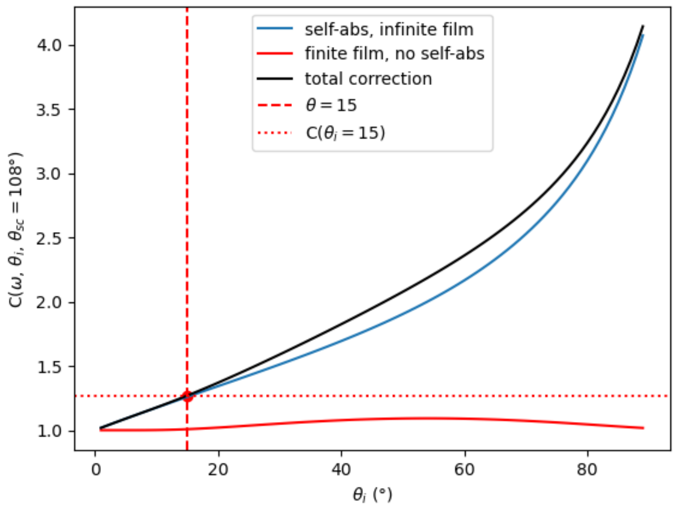

Fig. R3: Self-absorption correction for a 60 nm thin film for scattering angle 108°, zero energy lost in the scattering even, and assuming $\sigma\sigma'$ scattering channel. Attenuation length has been assumed to be 60 nm.

2. All measurements are on STO-grown, twinned thin films lacking chain order. Strain, surface fields, or twinning could stabilize or bias a super-lattice distinct from bulk behavior. No bulk crystals, thickness dependence, or alternative substrates are tested, leaving the generality of the observations unclear.

Authors: The comment of the Referee is that the generality of the superstructure observation could be strengthened by observations in films with other substrates or in bulk crystals.

Our films ($Pr_{0.3}Y_{0.7}Ba_2Cu_3O_7$, $Pr_{0.3}Y_{0.7}Ba_2Cu_3O_{6.67}$ and $PrBa_2Cu_3O_7$) have thicknesses higher than 60 nm. At these values, all the films are expected to be relaxed. As such, variations of substrates are not expected to make a difference. In fact, our choice of film thickness was made exactly to avoid substrate dependent results (and also to maximize signal from GI-XRD). Moreover, we point out that Ref. 36 (Ruiz et al.) reported the Q_1 order (although interpreted as charge order) on a single, twinned (and therefore on average tetragonal as our films) crystal of $Pr_{0.3}Y_{0.7}Ba_2Cu_3O_7$. The experimental observations of the peaks observed in this article are generally consistent with ours. In this fashion, the superstructure peak is observed in both crystals and films, and we believe that this fact rules out the scenario where its existence is solely determined by spurious effects due to the substrate.

3. The claim that Q1 suppresses pairing is highly speculative. There is no systematic mapping across Tc, nor field-tuned data to reveal canonical CDW-SC competition. The superconducting implications are asserted more strongly than the presented data warrant. The authors should elaborate more on this aspect.

Authors: We have revised the abstract so that the superstructure found in PBCO associated with the unusual electronic properties. In this fashion, there is no longer any statement made on competition with superconductivity.

4. The authors state that the Q1 reflection is "resolution-limited" but do not provide deconvolved in-plane or out-of-plane correlation lengths or the instrumental resolution function. Providing this data would allow a quantitative assessment of the spatial extent of the order.

We thank the Referee for this comment. The effective resolution depends on several experimental factors (i.e. monochromaticity of the beam, pixel size, divergence of the beam) and on the reconstruction procedure, that assigns (H,K,L) to every pixel in the detector as a function of rocking angle ω. We have taken the Bragg peaks of $SrTiO_3$, which is a commercial substrate of high quality and crystallinity, as a measure of the total experimental resolution. By fitting 24 peaks in $SrTiO_3$ (H,K,0) with $|H|,|K| \leq 2$ (excluding the (0,0,0) reflection) we have determined an effective resolution of 0.016 ± 0.005 r.l.u..

For REXS, the main contribution to the resolution comes from the finite size of the detector, an AXUV100-type photodiode (10mm x 10mm). Given the distance from the sample (50 cm) this gives an aperture of the scattering angle of $\sim 1°$, and a momentum resolution on H at the Q1 reflection (0.33,0,1) of 0.005 r.l.u.. We have now added a new section “Experimental resolution” in the Supplementary Material. Additionally, we now discuss the correlation length of the superstructure after deconvolution in the main text:

“*The correlation length of the Q_1 reflection also appears to be temperature independent. After estimation of the experimental resolutions of the GI-XRD and REXS (see Supplemental Material), we extract a momentum full-width-at-half-maximum (FWHM) of approximately 0.015 (0.01) r.l.u. for GI-XRD (REXS) for $Y_{0.7}Pr_{0.3}Ba_2Cu_3O_{6.67}$, and of 0.022 (0.014) r.l.u. for $PrBa_2Cu_3O_7$. The discrepancy between the two sets of measurements can be explained by the fact that GI-XRD*

probes the whole volume of the films, while REXS is limited to a spot size of 100×50 μm. This corresponds to a spatial correlation length ξ ≃ 2/FWHM Å ≈ 170 Å using the estimate from REXS."

5. While possibly beyond the scope of the present manuscript, independent cross-validation through spatially-resolved techniques such as STM or STEM could really strengthen the evidence for a true Pr-related order. Their absence weakens confidence in the interpretation.

Authors: Generally, we agree with the Referee that STM and STEM experiments would be valuable to further assess the microscopic origin of the Pr-induced lattice modulation. However, such characterizations are not straightforward in the present case: the relatively low Pr concentration makes it challenging to obtain cleaved surfaces suitable for STM, and detecting Pr-site modulations in STEM requires careful optimization of sample preparation and imaging conditions. At present, this investigation goes, as also stressed by the Referee, beyond the scope of this work. All our experimental evidence strongly supports a structural origin of the Q1 peak and does not indicate any electronic order, in contrast to what was claimed in previous works. For the future, we concur that STEM or related electron microscopy techniques would represent the most direct and informative extension of our study, but we leave this important effort for future studies.

6. The peak shown in Fig. 2(f) at the out-of-plane Cu-L resonance for Y0.7Pr0.3Ba2Cu3O6.7 appears weak. Can the authors clarify whether it is statistically significant? The authors should also comment on possible effects of fluorescence background on the resonance profiles, particularly for weak reflections like Q2?

Authors: We thank the referee for this remark. Indeed, the peak shown in Fig. 2f for the out-of-plane resonance is at the limit of our experimental sensitivity. The fitting has been obtained using a model comprising a linear background and a Gaussian peak. The width and centre of the Gaussian peak have been fixed to the values obtained by fitting the spectrum at the Cu resonance. To verify that the addition of a non-zero peak is necessary to model the data, we proceeded in two ways. First, we have verified that the 2σ ($\sim 95\%$) confidence interval of the gaussian peak area remains strictly above zero. In particular, we obtain $A = (3.6 \pm 1.35) \cdot 10^{-5}$. Secondly, we have performed a p-value test on the null model (just a linear background, which properly fits the dataset at the Pr energy 930.95 eV, yellow label in Fig.2) and a model of a linear background with the addition of a Gaussian peak. We have assumed that the experimental points have an independent Gaussian noise $N(0, \sigma_N^2)$ and estimated $\sigma_N \sim 3 \cdot 10^{-6}$ by calculating the standard deviation in the part of the spectrum far from the Q2 reflection (in the H range [0.20, 0.25] r.l.u.) after subtraction of the linear background. According to the Wilks theorem, the test statistics $-2\log(\Lambda) = \frac{1}{\sigma_N^2}\sum(x_i - \mu)^2$ can be modelled by a $\chi^2(k)$ distribution under the null model, with k equal to the number of additional parameters. Therefore, the probability that the null model is correct is $p = 1 - \chi^2(-2\log(\Lambda), k)$ where k=1 in our case since we have fixed the peak center and width to the one obtained at the main Cu resonance, where the peak is evident (middle panel of Fig.3f). We obtain $p = 0.0042$ for the REXS scan mentioned by the Referee, and $p \sim 6 \cdot 10^{-12}$ for the scan at the Cu resonance (which instead has 3 additional free parameters compared to

the null model since in this case we do not fix energy and width). Therefore, we are confident that a peak is present in the experimental spectra above Cu resonance. As a sanity check, taking the scan in the top of panel (f), where no peak is discernible, we obtain an amplitude of the peak of $0 \pm 0.015 \cdot 10^{-5}$ and a p-value of exactly 1 (not peak required with absolute certainty).

**Energy**	**Amplitude (arb. units)**	**p-value**	**K (free params)**
930.95 eV	0 ± 0.0155	1	1
932.45 eV	0.102 ± 0.013	$6 \cdot 10^{-12}$	3
933 eV	0.038 ± 0.013	0.0042	1

7. Have the authors quantified the uncertainty in δ1 and δ2 beyond the Gaussian fits? In Fig. 3(c), the scatter between non-resonant and resonant measurements suggests that the true uncertainty may be larger than the standard errors from the fits (for example, the 100 K non-resonant value differs noticeably from the resonant measurements). Since a small difference in the in-plane periodicity is used to distinguish Q1 and Q2, a more comprehensive statistical analysis that accounts for systematic uncertainties would strengthen the claim. Demonstrating reproducibility across multiple Brillouin zones or different experimental runs would further increase confidence. While I understand the time limitations for synchrotron experiments, I would urge the authors to consider this matter carefully.

We thank the Referee for this remark. We have now improved our estimation of Q_1 wavevector and of its uncertainty from high-energy GI-XRD by fitting the peaks across multiple Brillouin zones. Indeed, the uncertainty is higher than the one extracted from confidence intervals of the fitting. We attribute this spread of data to errors coming from the reconstruction algorithm, that assigns (H,K,L) coordinates to each pixel detector for each value of the rocking angle ω and the bins all the detectors images into a single 3D dataset. We have estimated a more reliable error by using the Birge method [Birge, R. T., Physical Review, **40**(2), 207–227 (1932)], which is the method of choice in case of inconsistent datasets. It scales the uncertainty over the mean of the independent measurements by a factor that depends on their likelihood function. A detailed explanation is reported in a new, dedicated section in the Supplemental Material ("Fitting of Q1 order in several Brillouin zones"). We have correspondingly modified Fig.3c, inserting the new estimate of Q1 coming from GI-XRD. Now, the uncertainty seems to be comparable to the real spread of data, while the measurements are still consistent with the REXS dataset.

8. Minor points:

There is a potential mislabeling in Fig. (b): Q1(blue) should be Q2?

Authors: We thank the Referee for spotting the mistake in Fig.2b. We have corrected the panel.

The resistivity curve for $Y_{0.7}Pr_{0.3}Ba_2Cu_3O_7$ seems noisy with some residual conductivity below Tc. While this is possibly due to a 2-probe measurement, please comment on it in text.

Authors: All transport measurements on the films discussed in the manuscript were performed in a four-probe Van der Pauw configuration. For the specific curve mentioned by the Referee, one of the four bonding wires was slightly loose, which accounts for the additional noise visible both

in the superconducting and in the normal state. However, it is important to stress that the zero-resistance critical temperature of the film is $T_c^0 = 45$ K, i.e., below this temperature there is no residual conductivity. In fact, most of the data points measured with the PPMS below T_c^0 yield negative values, i.e., a vanishing resistance within the experimental resolution.

Ref. 6 in SI is improperly formatted.

Authors: We thank the Referee for this comment. We have now corrected the reference.

Reviewer #3 (Remarks on code availability):

Not applicable.

Reply to Referee comments:

Reviewer #1

The authors have provided a thorough and persuasive response to the concerns raised in the initial review. In particular, the primary critique regarding the novelty of the work has now been convincingly addressed. By clearly distinguishing the structural nature of the Q1 order from previously reported electronic modulations, the authors offer an important clarification and refinement of the existing literature.

I have one remaining point for consideration. Given that the observed ordering phenomena appear to be highly sensitive to film quality and growth conditions—such as strain and defect density—it would be useful to clarify to what extent the proposed phase diagram can be regarded as universal for the $Y_{1-x}Pr_xBa_2Cu_3O_{6+y}$ system rather than being specific to thin-film realization.

This point does not undermine the overall impact or significance of the study. I therefore recommend the manuscript for publication in Nature Communications.

We sincerely thank the Referee for recommending our manuscript for publication.

We believe that the phase diagram sketched in Fig.4 can be considered general for the Pr-doped YBCO family. Indeed, despite some discrepancies with the current existing literature (Refs.36 and 37 in the manuscript), the core phenomenology that we observed is compatible with the films (Ref.37) and single crystals (Ref.36) investigated in previous studies. Additionally, our films have thicknesses above 60 nm and are therefore completely relaxed, minimizing the strain effects coming from the substrate. We have added a sentence in the "Discussion" section to highlight our view:

"In general the broad phenomenology is similar in the three studies. Therefore we believe that, despite discrepancies which might be related to differences in sample type (thin films vs single crystals), the core phase diagram presented in Fig.4 is generally valid for the $Pr_xY_{1-x}Ba_2Cu_3O_{6+y}$ system."

Reviewer #2

As mentioned in my previous report, this work presents significant advances in understanding the physics of charge orders in the YPrBCO system. The authors have carefully and thoroughly addressed my comments as well as those of the other referees, and have revised both the manuscript and the Supplementary Materials accordingly.

The revised version provides additional and clearer insights into the phase diagram of Pr-doped YBCO, extending and clarifying previous studies (Refs. 36 and 37). I therefore conclude that the

revised manuscript meets the standards for publication in Nature Communications, and I recommend acceptance as is.

We sincerely thank the Referee for recommending our manuscript for publication.

Reviewer #3

I thank the authors for their careful and detailed responses and for the substantial revisions to the manuscript and Supplementary Material. Overall clarity, statistical analysis, and the balance of interpretation have improved significantly.

Most of my major concerns have been addressed. The discussion of site-specific resonances is now more appropriately nuanced, explicitly acknowledging possible overlap between out-of-plane Cu and Pr-related states. The expanded treatment of instrumental resolution, correlation lengths, and uncertainty estimation across multiple Brillouin zones strengthens the experimental basis of the conclusions.

Some limitations however remain. While the distinction between Q1 and Q2 is now better supported statistically, the weakest resonant features, particularly at the out-of-plane Cu-L resonance, remain close to the experimental sensitivity limit. Statistical analysis supports it's presence, but the significance depends on fixed peak parameters and background assumptions and should therefore be interpreted with appropriate caution. More generally, the microscopic origin of the Q1 superstructure is inferred indirectly from resonant behavior and scattering geometry. Independent real-space or site-resolved probes would provide a valuable cross-check, though this lies beyond the scope of the present work.

Overall, the revised manuscript presents a technically strong and conceptually clearer differentiation between two closely related ordering phenomena in Pr-substituted YBCO. With the more careful framing now adopted by the authors, I believe the work is suitable for publication.

We sincerely thank the Referee for recommending our manuscript for publication.

We agree with the Referee that the use of independent techniques could help in shedding light on the microscopic origin, and on the orbital content, of the Q1 order. We have now added a sentence at the end of the manuscript to give an outlook on the subsequent steps:
"In perspective, although Scanning Tunneling Microscopy remains challenging for these samples (and in general for the REBCO family), progress in surface preparation and termination control could make real-space, site-sensitive measurements increasingly accessible, further clarifying the microscopic origin of the observed Q1 order.".
